# Development and implementation of rapid metabolic engineering tools for chemical and fuel production in *Geobacillus thermoglucosidasius* NCIMB 11955

**DOI:** 10.1186/s13068-016-0692-x

**Published:** 2017-01-03

**Authors:** Lili Sheng, Katalin Kovács, Klaus Winzer, Ying Zhang, Nigel Peter Minton

**Affiliations:** Clostridia Research Group, BBSRC/EPSRC Synthetic Biology Research Centre (SBRC), School of Life Sciences, University of Nottingham, University Park, Nottingham, NG7 2RD UK

**Keywords:** Allelic exchange, In-frame deletion, Counter-selection marker, *pyrE*, *Geobacillus thermoglucosidasius*, Whole-genome sequencing

## Abstract

**Background:**

The thermophile *Geobacillus thermoglucosidasius* has considerable attraction as a chassis for the production of chemicals and fuels. It utilises a wide range of sugars and oligosaccharides typical of those derived from lignocellulose and grows at elevated temperatures. The latter improves the rate of feed conversion, reduces fermentation cooling costs and minimises the risks of contamination. Full exploitation of its potential has been hindered by a dearth of effective gene tools.

**Results:**

Here we designed and tested a collection of vectors (pMTL60000 series) in *G. thermoglucosidasius* NCIMB 11955 equivalent to the widely used clostridial pMTL80000 modular plasmid series. By combining a temperature-sensitive replicon and a heterologous *pyrE* gene from *Geobacillus kaustophilus* as a counter-selection marker, a highly effective and rapid gene knock-out/knock-in system was established. Its use required the initial creation of uracil auxotroph through deletion of *pyrE* using allele-coupled exchange (ACE) and selection for resistance to 5-fluoroorotic acid. The turnaround time for the construction of further mutants in this *pyrE* minus strain was typically 5 days. Following the creation of the desired mutant, the *pyrE* allele was restored to wild type, within 3 days, using ACE and selection for uracil prototrophy. Concomitant with this process, cargo DNA (*pheB*) could be readily integrated at the *pyrE* locus. The system’s utility was demonstrated through the generation in just 30 days of three independently engineered strains equivalent to a previously constructed ethanol production strain, TM242. This involved the creation of two in-frame deletions (*ldh* and *pfl*) and the replacement of a promoter region of a third gene (*pdh*) with an up-regulated variant. In no case did the production of ethanol match that of TM242. Genome sequencing of the parental strain, TM242, and constructed mutant derivatives suggested that NCIMB 11955 is prone to the emergence of random mutations which can dramatically affect phenotype.

**Conclusions:**

The procedures and principles developed for clostridia, based on the use of *pyrE* alleles and ACE, may be readily deployed in *G. thermoglucosidasius*. Marker-less, in-frame deletion mutants can be rapidly generated in 5 days. However, ancillary mutations frequently arise, which can influence phenotype. This observation emphasises the need for improved screening and selection procedures at each step of the engineering processes, based on the generation of multiple, independent strains and whole-genome sequencing.

**Electronic supplementary material:**

The online version of this article (doi:10.1186/s13068-016-0692-x) contains supplementary material, which is available to authorized users.

## Background

The continued utilisation of fossil fuels for energy and chemical generation is not tenable. A finite resource, their extraction, processing and use causes environmental damage and pollution. One option is to use microbial fermentative processes to generate the chemicals and fuels that the society and industry need from sustainable, lignocellulosic feedstocks. In the case of bioethanol production, the most used microbial process organism is yeast [1]. Other microbial species are, however, being pursued, including thermophilic bacteria belonging to the genus *Geobacillus.* These aerobic or facultative anaerobic bacteria are able to ferment a wide range of sugars (glucose, cellobiose, xylose and mixtures of glucose, xylose, and arabinose), typical of those found in lignocellulosic substrates, and can grow over a wide range of temperatures between 40 and 70 °C [2]. Higher growth temperatures improve the rate of feed conversion and make the process more effective by reducing cooling costs during fermentation. They also reduce the risk of contamination by other microorganisms as well as conferring desirable properties on the growth medium, such as reduced viscosity, reduced energy requirements for mixing and increased diffusion rates and substrate solubility [3]. *Geobacillus thermoglucosidasius* has previously been engineered for industrial bioethanol production from lignocellulosic feedstock [4]. The quantities of ethanol obtained from the engineered strain, TM242, ranged from 80 to 95% of theoretical yields with maximum productivity being attained with cellobiose as a carbon source. The study demonstrated that TM242 was capable of effective simultaneous saccharification and fermentation and the rapid metabolism of the range of sugars typically found in hydrolysates of biomass.

The engineering of microbial production strains is reliant on the availability of effective gene tools that may be used to bring about the requisite changes to metabolic pathways through gene ‘knock-out’ (KO) and ‘knock-in’ (KI). In the case of *G. thermoglucosidasius*, the tools developed allowed the generation of strain TM242 through the sequential deletion of the *ldh* gene (encoding lactate dehydrogenase) and *pfl* gene (coding for pyruvate formate lyase) and substitution of the promoter of the *pdh* gene (encoding pyruvate dehydrogenase) with a stronger promoter [4]. Although adequate, their successful implementation required an extensive screening process to isolate the desired double-crossover (DC) mutants. This involved numerous sub-cultures, firstly to isolate antibiotic (kanamycin, Km)-resistant (^R^), single-crossover plasmid integrants and secondly to isolate DC allelic exchange mutants which became Km sensitive (^S^). The latter stage involved replica plating of single colonies onto agar media with and without Km, to identify clones in which the integrated plasmid, and the antibiotic resistance gene (*kan*) it carried, had excised and been lost. Then, to distinguish between those cells that had reverted to wild type (WT) and the desired allelic exchange mutant, metabolic profiling was undertaken. Finally, confirmation that the clone identified contained the desired mutant allele was derived using an appropriate PCR assay.

One way to increase the efficiency with which the desired DC mutants can be isolated is through the use of a vector-encoded, counter-selection (or negative selection) marker [5]. Cells carrying a plasmid, autonomous or integrated, which incorporate a counter-selection marker, are unable to grow in the presence of the counter-selection agent, a consequence of the conversion of this agent to a toxic metabolite through the action of the marker-encoded factor. It follows that if single-crossover (SC) integrants are plated on media containing the counter-selection agent, only those cells in which the plasmid has excised, and subsequently been lost, can grow. Such cells will be composed of two types of cell lines: those in which the excision event has resulted in the original parental strain, or those in which the parental allele has been exchanged with the intended mutant allele. The two types of cell can be distinguished by an appropriate PCR-based assay.

Numerous counter-selection markers have been employed to facilitate the isolation of DC mutants [5, 6]. Of widespread utility are those genes involved in uracil metabolism, and in particular *pyrE* which encodes orotate phosphoribosyltransferase [7, 8]. It may be used as a positive or a negative selection marker as its presence is essential in the absence of exogenous pyrimidines and renders 5-fluoroorotic acid (5-FOA) toxic to cells. The latter is a consequence of the conversion of 5-FOA to 5-fluorouridine monophosphate (5-FUMP). This approach has been exploited in a number of bacteria for marker-less deletions [9–13], including the thermophiles *Clostridium thermocellum* [14] and *Geobacillus kaustophilus* [15].

In order for *pyrE* to be used as a counter-selection marker, it is crucial that the host lacks orotate phosphoribosyltransferase activity. The necessary *pyrE* mutant may be rapidly generated using allele-coupled exchange (ACE), a special form of allelic exchange [16]. Crucially, the design of the ACE-created *pyrE* mutant strain is such that its *pyrE* allele can be rapidly (3 days) restored to wild type (WT) using an appropriate ACE correction vector allowing the specific in-frame deletion mutant to be characterised in a clean, otherwise WT background [17, 18]. Moreover, cargo DNA can be chromosomally integrated along with the restoration of *pyrE* gene, allowing for the integration of heterologous DNA. Exploitation of *pyrE* alleles and ACE form the basis of a roadmap for developing gene systems in *Clostridium* species [6].

By exploiting *pyrE* alleles as both negative and positive selection markers, we established a suite of recombination-based genetic tools that can be used to rapidly engineer the metabolism of *G. thermoglucosidasius* NCIMB 11955 by both KO and KI. Marker-less, in-frame deletion mutants can be rapidly generated in 5 days. Integration of DNA cargo is accomplished in just 3 days (Additional file 1: Figure SI). As an exemplification of the method, strains equivalent to the industrial production strain, TM242 (two deletions and a promoter replacement), could be reproducibly generated in 30 days. However, ancillary mutations frequently arise in this strain, which can influence phenotype.

## Results

### Construction of modular *Geobacillus* vectors

**Fig. 1:**
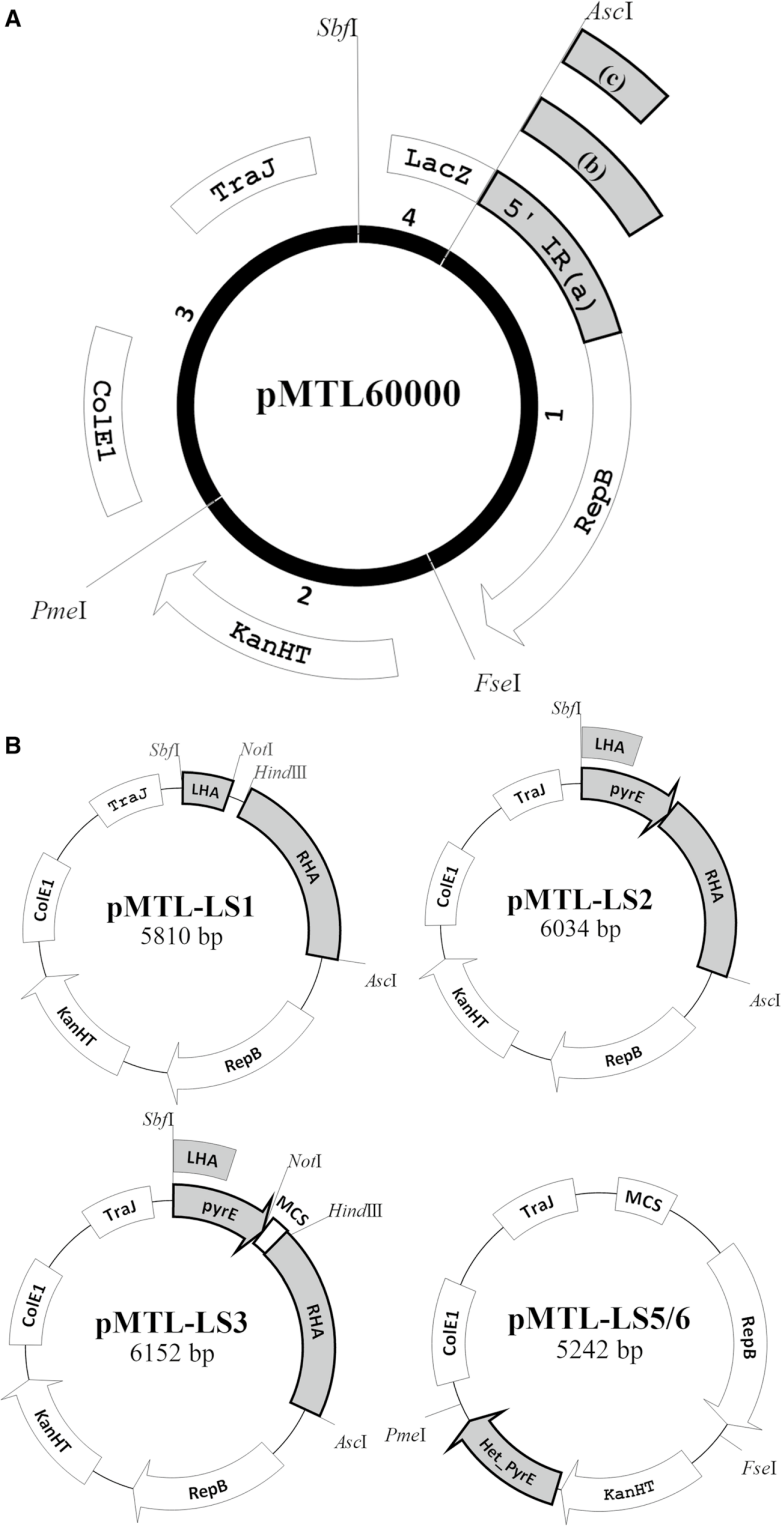
Plasmids used in this study. All plasmids used in this study are based on the pMTL60000 modular shuttle vector series (**A**), defined by four modules (Gram-positive replicon (*1*), *Asc*I/*Fse*I, selectable marker (*2*), *Fse*I/*Pme*I, gram-negative replicon (*3*), *Pme*I/*Sbf*I, application-specific module (*4*), *Sbf*I/*Asc*I), with the corresponding components being *S. aureus* pUB110 replicon, thermostable kanamycin adenyltransferase gene (KanHT) conferring kanamycin resistance, *ColE*I origin of replication and the *oriT*-*traJ* mobilisation region, and LacZα MCS. The 5′ incompatible region of RepB (5′ IR) is reduced from 412 bp (*a*) to 362 bp (*b*) and to 189 bp (*c*) to give three variants of pMTL61110 (4809 bp), pMTL62110 (4591 bp) and pMTL63110 (4418 bp), respectively. Plasmids for *pyrE*-based allele-coupled exchange (ACE) are all derived from pMTL62110 (**B**). Insertion of a 299-bp internal *pyrE* sequence between *Sbf*I and *Not*I (LHA) and a 1200-bp *pyrE* downstream sequence between *Hin*dIII and *Asc*I gives the *pyrE* knockout vector pMTL-LS1. Insertion of a 1838-bp sequence consisting of the entire *pyrE* sequence lacking only the start codon and the same downstream RHA between *Sbf*I and *Asc*I gives the *pyrE* repair vector pMTL-LS2. Replacement of the LHA of pMTL-LS1 with the entire *pyrE* gene lacking only the start codon (639 bp) gives the *pyrE* integration vector, pMTL-LS3. DNA to be integrated chromosomally is inserted at the multiple cloning site. Fusion of KanHT with a heterologous *pyrE* (*G. kaustophilus*; *G. thermoleovorans*) gives deletion vectors pMTL-LS5 and pMTL-LS6. Homology arms of the desired knockout genes are cloned at the MCS

To standardise plasmid construction, the modular approach of Heap et al. [19] was adopted (Fig. 1). Plasmid pMTL61110 was assembled using the *Staphylococcus aureus kan* gene (encoding resistance to kanamycin, Km) as the selection marker module (*Fse*I–*Pme*I restriction fragment) and the pUB110 replicon as the Gram-positive replication origin module (*Asc*I–*Fse*I restriction fragment). Both components have previously been shown to function in *G. thermoglucosidasius* [20]. In addition, the plasmid incorporated a Gram-negative replicon module, defined by the ColE1 origin of replication and the *oriT*–*traJ* mobilisation region (flanked by restriction enzyme recognition sites for *Pme*I and *Sbf*I), and an application-specific module containing a multiple cloning site (MCS) derived from pMTL81551 (flanked by restriction enzyme recognition sites for *Sbf*I and *Acs*I). The plasmid generated was shown to successfully transform *G. thermoglucosidasius,* selecting for Km^R^ colonies, at frequencies of 10^3^–10^4^ colonies per µg, which were comparable to the commonly used shuttle vectors for *G. thermoglucosidasius* [21, 22].

 If the frequency of transformation of a particular organism is sufficiently high, then KO and KI mutants can be most easily generated using suicide vectors that are unable to replicate in the recipient cell. In these instances, DC mutants may be directly selected. In the absence of the requisite high frequency of transformation, the required allelic exchange mutants are generated through the sequential isolation of a SC integrant, followed by the isolation of the DC mutant following plasmid excision. Both steps are facilitated by the use of defective plasmid replicons. In this approach, termed ‘pseudo-suicide’ [23], integrated vectors endow a growth advantage on the cell in the presence of antibiotic over cells carrying the autonomous vector. As SC integrants grow faster, they produce visibly larger colonies. Moreover, following plasmid excision, pseudo-suicide plasmids are more rapidly lost from the cell.

The DNA fragment encompassing the pUB110 replicon present in the vector pMTL61110 is equivalent to that previously used in plasmid pTMO31 [4]. Segregational stability studies undertaken on *G. thermoglucosidasius* NCIMB 11955 (from here on referred to as 11955) demonstrated that in the absence of antibiotic selection, 70% of the cells retained the plasmid (Table 1), an equivalent segregational instability to pTMO31 and pNW33N. In order to derive a more unstable plasmid, we elected to test the effect of foreshortened variants of the pUB110 replicon (Additional file 1: Figure SII). It has previously been reported that a 358-bp incompatibility region (IncA) that resides 5′ to the pUB110 *repB* gene acts as a trans-acting element involved in the control plasmid replication [24]. Accordingly, two derivative plasmids were constructed in which the 412-bp region preceding *repB* present in plasmid pMTL61110 was reduced to 362 and 189 bp. The plasmids generated were denoted pMTL62110 and pMTL63110, respectively (Fig. 1). Both of the new plasmids exhibited a significant increase in segregational instability (Table 1) with less than 10% of the cells retaining the plasmid after 72 h (Additional file 1: Figure SIII). These data indicated that the region between position −412 and −362 relative to *repB* plays a role in plasmid segregational stability.

**Table 1:** Plasmid segregational stability

Plasmid	Retainment per generation	Loss per generation
pTMO31	0.995 ± 1.40 × 10^−3^	5.23 × 10^−3^ ± 1.40 × 10^−3^
pNW33N	0.999 ± 1.93 × 10^−4^	9.59 × 10^−4^ ± 1.93 × 10^−4^
pMTL61110	0.997 ± 4.37 × 10^−4^	2.59 × 10^−3^ ± 4.37 × 10^−4^
pMTL62110	0.976 ± 4.68 × 10^−3^	2.39 × 10^−2^ ± 4.68 × 10^−3^
pMTL63110	0.965 ± 5.33 × 10^−3^	3.46 × 10^−2^ ± 5.33 × 10^−3^

It has been suggested that the pUB110 replicon is temperature sensitive and does not function at 65 °C and above [25]. Additionally, the pUB110 *kan* gene does not confer resistance to Km above 60 °C [26]. To test the temperature sensitivity of the modular shuttle vectors, derivatives of strain 11955 harbouring the three plasmids were grown at temperatures between 52 and 60 °C in media supplemented with 12.5 μg/ml Km. Cells harbouring pMTL61110 were able to grow up to 60 °C on both TSA and 2SPYNG agar media. Cells carrying either pMTL62110 or pMTL63110 (incorporating the shorter replicon fragments) were only able to grow up to 55 °C, and not at 60 °C, a phenotype that should prove useful in selecting for plasmid loss.

### Generation of a *G. thermoglucosidasius* 11955 Δ*pyrE* mutant using ACE

**Fig. 2:**
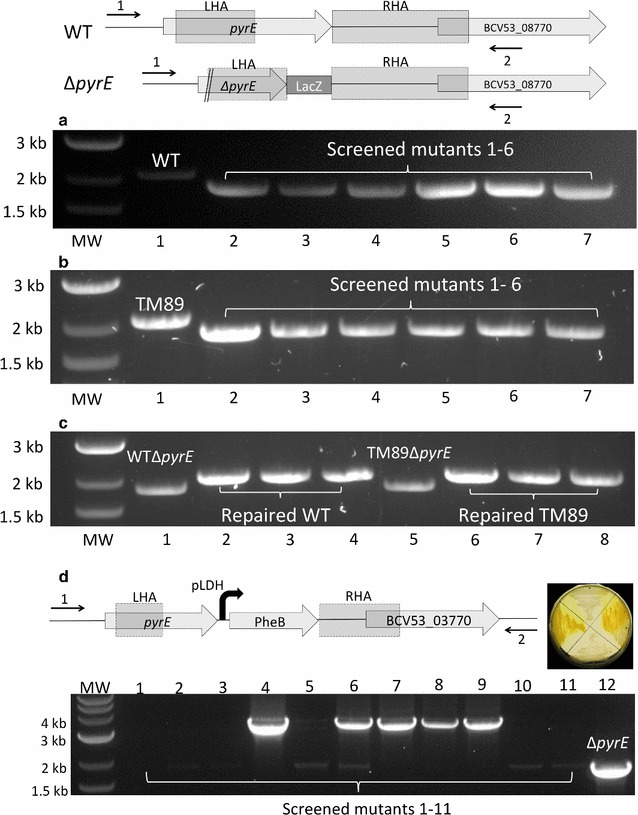
Screening of *pyrE* knockout, *pyrE* repair and integration at the *pyrE* locus. *PyrE* orotate phosphoribosyltransferase; BCV53_03770: transcriptional regulator, *PheB* G. *stearothermophilus* 2,3-dioxygenase, *LHA* left homology arm (299 bp), *RHA* right homology arm (1200 bp), *MW* 2-log DNA marker (NEB), molecular weight marker, *WT G. thermoglucosidasius* NCIMB 11955 wild type, Δ*pyrE* G. *thermoglucosidasius pyrE* knockout mutant. The chromosomal region at the *pyrE* locus is illustrated for WT and Δ*pyrE*. All screening was conducted using primers pyrE_C1_F (*1*) and pyrE_C2_R (*2*), giving the expected PCR product of 2101 bp for WT, 1876 bp for Δ*pyrE* and 3490 bp for the integration of *pheB*. *Lanes 2*–*7* (**a**) and *1*–*12* (**b**) represent the screened DNA samples from randomly selected uracil auxotrophic colonies for WT (**a**) and TM89 (**b**). *Lanes 2–4* and *6*–*8* (**c**) represent the screened DNA samples from randomly selected uracil prototrophic colonies derived by repairing of *pyrE* with pMTL-LS2 in both WTΔ*pyrE* and TM89Δ*pyrE*. *Lanes 1–12* (**d**) represent the screened DNA samples from randomly selected uracil prototrophic colonies derived from the integration of *pheB* at the *pyrE* locus. Of the 11 screened colonies, 5 had the expected PCR band size. Correct integrants are plated on TSA and sprayed with catechol for phenotypic validation (**d**)

Prior to the generation of the *pyrE* deletion mutant, it was important to establish the minimal media that could be used to both select (5-FOA^R^) and confirm the *pyrE* phenotype (uracil auxotrophy). Fortuitously, 11955 was found to grow on clostridial basal media (CBM) supplemented with 1% xylose (CBM1X) and that the minimal inhibitory concentration (MIC) for 5-FOA was 300 µg/ml. Having established the most defective Gram-positive replicon, a *pyrE* KO allele-coupled exchange (ACE) vector (pMTL-LS1) equivalent to the clostridial vector pMTL-JH12 [16] was constructed by cloning the requisite strain 11955-specific LHA and RHA regions from the *pyrE* locus into plasmid pMTL62110 (Fig. 1). Following the transfer of pMTL-LS1 into 11955, SC integrants were selected based on faster growing, larger colonies. Such integrants had invariably integrated via the longer right homology arm (RHA), due to its greater size (1200 bp) compared to the shorter (300 bp) left homology arm (LHA). These faster growing colonies were cultivated in 2SPYNG media at 52 °C before plating onto CBM1X agar supplemented with 5-FOA (300 µg/ml) and uracil (20 µg/ml). The 5-FOA^R^ colonies that developed were then replica plated onto CBM1X media with and without uracil to confirm uracil auxotrophy. The authenticity of the putative *pyrE* mutants was then confirmed by PCR amplification of the mutant allele using flanking primers and Sanger sequencing of the amplified DNA fragment. Out of 6 5-FOA^R^ colonies screened, all proved to be authentic mutants. To confirm the reproducibility of the protocol, the same procedure was carried out on TM89 (an *ldh* mutant [4]) and pure TM89*ΔpyrE* strains were also readily isolated (Fig. 2). The mutants exhibited normal growth rates when cultivated in rich 2SPYNG medium (Fig. SIV) but required at least 10 µg/ml uracil supplementation to achieve equivalent growth rates to the WT on CBM1X medium (Additional file 1: Table SI).

### Restoration of the *ΔpyrE* allele to wild type and concomitant introduction of cargo DNA

In keeping with the roadmap principles [6], two ACE vectors were generated: an ACE correction vector designated pMTL-LS2, equivalent to pMTL-ME6, and a pMTL-ME6C equivalent vector pMTL-LS-3 (Fig. 1) [18]. The former carried a single, contiguous region (1500 bp) of homology to the *pyrE* gene and the region downstream, while the latter carried the same region but included a segment of DNA incorporating multiple cloning sites (MCS) between the LHA (essentially the *pyrE* gene) and the RHA (the 1200-bp region 3′ to *pyrE*). Both plasmids were shown to be able to rapidly restore the 11955 *pyrE* mutant to prototrophy through ACE (Fig. 2). Thus, the re-streaking of primary Km^R^ transformants of either plasmid onto CBM1X agar lacking exogenous uracil resulted in discrete colonies that on examination with an appropriate PCR assay all carried the wild-type *pyrE* allele.

To demonstrate that the ACE vector pMTL-LS3 could be used to insert cargo DNA into the genome, the *pheB* reporter gene encoding catechol 2,3-dioxygenase [27] was cloned into the MCS region under the control of the promoter (P_ldh_) of the *G. stearothermophilus ldh* gene. Spraying of catechol (“Methods” section) to the correct colonies that arose on CBM1X minimal plates (5 out of 11), validated by PCR screening and Sanger sequencing, resulted in vivid yellow colouration indicative of the presence of catechol 2,3-dioxygenase (PheB) (Fig. 2). These results demonstrate that heterologous DNA can be integrated, and functionally expressed, at the *pyrE* locus of *G. thermoglucosidasius* using ACE.

### Complementation of the *G. thermoglucosidasius ΔpyrE*

Having created an appropriate *pyrE* mutant of NCIMB 11955 and TM89, a *pyrE*-based KO vector equivalent to those (pMTL-YN3/4 and pMTL-ME3, respectively) constructed for use in *Clostridium difficile* and *Clostridium acetobutylicum* [17, 18] was needed. An essential requirement was a functional, thermophilic *pyrE* gene to act as the counter-selection marker which, to avoid unintended homologous recombination events, should lack any substantive similarity to the chromosome of *G. thermoglucosidasius.* Accordingly, we compared the nucleotide similarity of the *G. thermoglucosidasius pyrE* gene to homologues in other thermophilic bacilli and choose the two with the lowest identity, namely the *pyrE* genes of *Geobacillus kaustophilus* DSM 7263 (75%) and *Geobacillus thermoleovorans* DSM 11667 (76%) (Additional file 1: Table SII). Each promoter-less gene was PCR amplified from its respective genome and combined through SOE PCR with a PCR-amplified DNA fragment encompassing the *kan* gene and its native promoter (“Methods” section). Through appropriate primer design, the final *kan*::*pyrE* cassette was flanked by *Fse*I and *Pme*I restriction recognition, enabling the SOE fragment to be cloned between the equivalent sites of pMTL62110, generating the plasmids pMTL-LS5 (*G. kaustophilus pyrE*) and pMTL-LS6 (*G. thermoleovorans pyrE*). The design was such that both *kan* and *pyrE* would be under the transcriptional control of the former gene’s promoter.

To confirm that the two *pyrE* genes were functional, plasmids pMTL-LS5 and pMTL-LS6 were transformed into strain 11955Δ*pyrE*, and the ability of the resultant transformants to grow on CBM1X without uracil supplementation was tested. Both plasmids restored the mutant to uracil prototrophy (Additional file 1: Figure SV). The effect on sensitivity to 5-FOA was also tested. As expected, whereas the 11955*ΔpyrE* strain could grow on media supplemented with 600 µg/ml of 5-FOA, the cells carrying either pMTL-LS5 or pMTL-LS6 could not (Additional file 1: Figure SVI). NCIMB 11955Δ*pyrE* transformants containing the vector control, pMTL62110, remained auxotrophic for uracil and resistant to 5-FOA. These data confirm that either heterologous *pyrE* gene may be used as a counter-selection marker in *G. thermoglucosidasius.* Plasmid pMTL-LS5 was chosen as the prototype KO vector.

### KO vector exemplification

To test the utility of pMTL-LS5k for gene KO, we chose the *trpB* gene as a target (encoding tryptophan *β*-synthase) due to its potential as another *pyrE* equivalent marker [tryptophan auxotrophy, 5-fluoroindole (FI) resistance] [28]. Accordingly, KO cassettes comprising equal-sized (500 bp) LHA and RHA were assembled by SOE PCR (“Methods” section) and cloned between the *Kpn*I and *Bam*H1 sites of pMTL-LS5 yielding the KO plasmid pMTL-LS5::*trpB*. It was transformed into 11955Δ*pyrE* together with the control plasmid pMTL-LS5. Transformants were selected on TSA plates supplemented with Km that were incubated at 52 °C. Following the development of colonies, they were streaked onto fresh plates and incubated at the non-permissive temperature, 60 °C. Only those cells carrying plasmids that possessed homology arms (pMTL-LS5::*trpB*) yielded discrete colonies on the re-streak plates. The re-streak of transformants harbouring the control plasmid pMTL-LS5 did not result in discrete colonies (Additional file 1: Figure SVII). This observation is consistent with the fact that autonomous plasmids carrying the derivatized, foreshortened pUB110 replicon cannot replicate at this temperature. The only cells that can grow are those in which vector integration has occurred, for which a region of plasmid homology is required.

The pMTL-LS5::*trpB* harbouring cells were then passaged once more at 60 °C on CBM1X agar lacking uracil and then subjected to a PCR screen. The two primers employed were complementary to the chromosome, external to the homology arm carried by the plasmid, and to the vector backbone (“Methods” section). The successful amplification of a DNA demonstrated that all of the colonies were composed of SC integrants, with recombination through either homology arm at the same frequency, 50%.

**Fig. 3:**
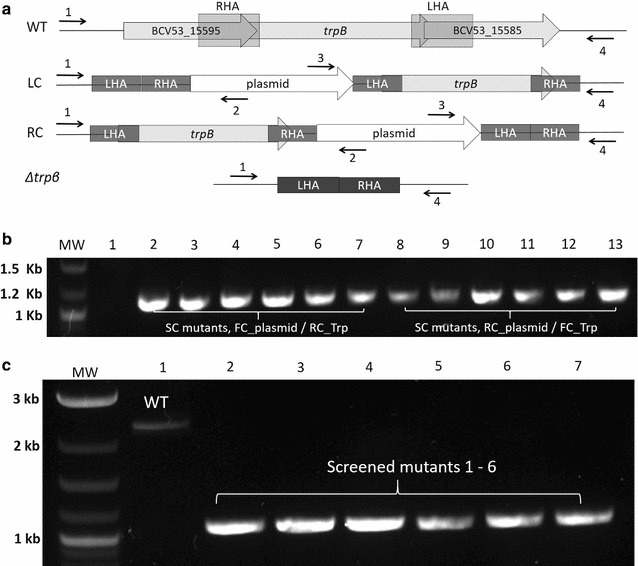
Screening of single and double-crossover mutant at tryptophan synthase beta locus. **a** A schematic representation of the two possible single crossover (SC) integrants, LC and RC. The former is where integration occurs at the 500 bp, left homology arm (LHA), while the latter is where integration is at the 500 bp, right homology arm (RHA). Illustrated genes are *trpB* tryptophan synthase beta, *BCV53_15585* indole-3-glycerol phosphate synthase, *BCV53_15595* tryptophan synthase alpha. The position and orientation of the four primers (1 to 4) used for PCR screening are shown above and below each represented region. **b** PCR screening of twelve single crossover integrants which have either integrated at the LHA (*lanes 2–7*) or the RHA (*lanes 8–13*). With the former, the amplified DNA fragment using primer 1 (FC_TRP) and primer 2 (RC_Plasmid) was 1125 bp, as opposed to 3005 bp if integration at the RHA had occurred. In the case of SC integrants where integration occurred at the RHA (*lanes 8–13*) the size of the DNA fragment amplified using primers 3 (FC_Plasmid) and 4 (RC_TRP) was 1171 bp as opposed to 2993 bp if integration had been at the LHA. *Lane 1* is the wildtype (WT) strain, which generates no DNA fragment with any primer combination. **c** Screening of putative double crossover (DC) mutants using primers 1 and 4. The WT (*lane 1*) generates a 2202 bp DNA fragment in PCR whereas DNA template from a Δ*trpB* mutant (*lanes 2–7*) generate a 1089 bp fragment. MW 2-log DNA marker (NEB) molecular weight marker

Having confirmed that all of the transformants obtained at 60 °C were pure single-crossover mutants, colonies were re-streaked on CBM1X supplemented with uracil (20 μg/ml), 5-FOA (300 μg/ml), tryptophan (1 μg/ml) and 5-FI (500 μg/ml). The concentration of 5-FI used (500 μg/ml) represented the determined MIC for this analogue for 11955. Discrete colonies were readily obtained following overnight incubation. PCR amplification and sequencing verification with primers on either side of the homology arms revealed that all of the 6 clones screened had the expected deletion (Fig. 3).

### Engineering strains for ethanol production

To demonstrate the utility of the developed method, we recreated the industrial bioethanol production strain TM242 from its parental strain, NCIMB 11955. Stepwise, the *ldh* gene (encoding lactate dehydrogenase) was first deleted in the 11955Δ*pyrE* background (LS001: Δ*pyrE,* Δ*ldh*), followed by the creation of a *pdh* (encoding pyruvate dehydrogenase) up-promoter mutation (replacement of its promoter with that of the *G. stearothermophilus* LDH promoter) (LS003: Δ*pyrE,* Δ*ldh, pdh* ^*up*^) and finally deletion of the *pfl* (encoding pyruvate formate lyase) gene (LS004: Δ*pyrE,* Δ*ldh, pdh* ^*up*^, Δ*pfl*). Lastly, the ACE correction vector pMTL-LS2 was used to repair the truncated *pyrE* gene in the triple deletion mutant LS004 to yield LS242 (Δ*ldh, pdh* ^*up*^, Δ*pfl*). In each step, no SC screening was required and each desired DC deletion mutation was consistently obtained. Typically, the DNA derived from 8 to 12 colonies was subjected to PCR and the amplification products were screened for the presence of a DNA fragment of both the correct size and expected nucleotide sequence. The generation of LS242 from the parental strain was accomplished within 30 days, demonstrating the rapidity of the method.

**Fig. 4:**
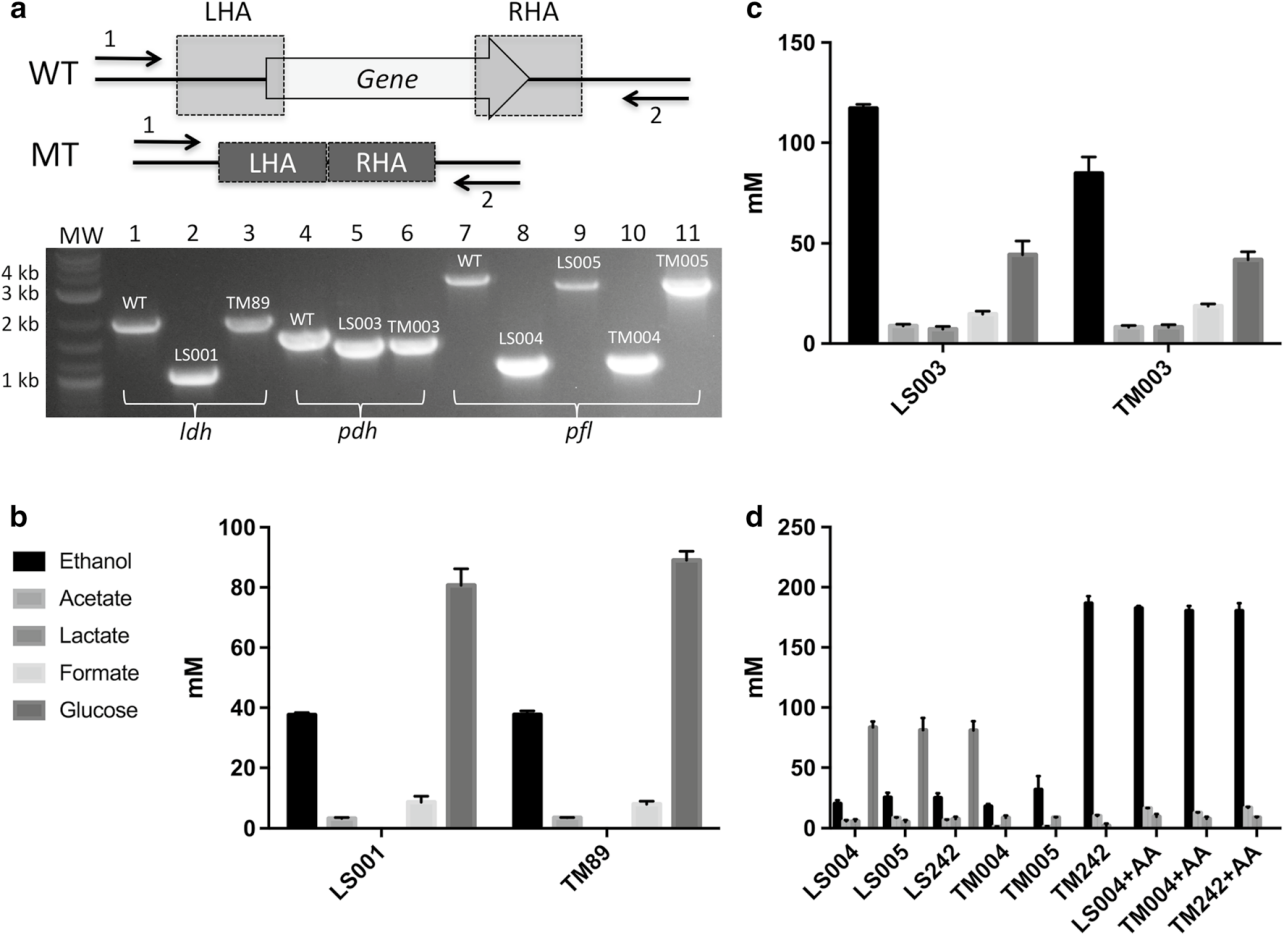
Fermentation profile of constructed strains. Mutants generated in this study are validated using PCR and Sanger Sequencing (**a**) before characterization (**b**–**d**). To achieve a clean in-frame deletion, typically homology arms (*left* and *right*) are designed to include only the start and stop codons of the desired knockout-target gene. Primers flanking just outside of the homology arms (*1*, *2*) are used for PCR screening. Lactate dehydrogenase (*ldh*) locus is screened with primers FC_LDH and RC_LDH (*lane 1*–*3*), with the expected size of 2031 bp for wild type (WT), 1130 bp for LS001 (Δ*ldh*) and 1981 bp for TM89 (*ldh* ^−^). Pyruvate dehydrogenase *(pdh)* locus is screened with primers FC_PDH and RC_PDH (*lane 4*–*6*), with the expected size of 1716 bp for WT and 1495 bp for promoter replacement with *G. stearothermophilus ldh* promoter (LS003, TM003). The pyruvate formate lyase (*pfl*) locus is screened with primers FC_PFL and RC_PFL (*lane 7*–*11*), with the expected size of 3348 bp for WT, 1122 bp for *pfl* deletion (Δ*pfl*) (LS004, TM004) and 2996 bp for *pfl* disruption (*pfl* ^−^) in the same context as that of TM242, involving a 360-bp central region disruption of *pfl* replaced by a *Not*I site (LS005, TM005). Solvent profiles (ethanol, acetate, lactate, formate and glucose) of the constructed strains, in three biological replicates, are characterised by HPLC after 24 h of fermentation using 40 ml of ASYE with 1% yeast extract and 2% glucose in a 50-ml Falcon tube at 60 °C. AA indicates the addition of 0.1 mM acetic acid

As our method allowed the precise excision of genes with high selectivity, we opted to create clean in-frame deletions (Δ*ldh* and Δ*pfl*), leaving only the start and stop codons intact. This differs from the described strategy used in the generation of TM242 [4], in which both genes were merely centrally disrupted through the incorporation of a *Not*I site. In the case of *pfl*, this caused premature termination of the PFL ORF from E332. However, the presence of an in-frame AUG start codon immediately downstream of the inserted *Not*I site meant that potentially a second ORF is present encoding a 302-amino acid protein. Consequently, the TM242 strain could potentially produce two proteins which may retain some residual PFL activity. Hence, from LS003, LS005 was constructed, which has an identical mutant *pfl* allele to TM242 (designated *pfl* ^−^ as opposed to Δ*pfl*). In addition, we also generated from TM89, the *ldh* ^−^ progenitor strain of TM242, all the equivalent strains, including TM003 (Δ*pyrE, ldh* ^−^, *pdh* ^*up*^), TM004 (Δ*pyrE, ldh* ^−^, *pdh* ^*up*^, Δ*pfl*) and TM005 (Δ*pyrE, ldh* ^−^, *pdh* ^*up*^ *, pfl* ^−^), with the latter strain having exactly the same three gene modifications that were present in TM242 (Fig. 4).

### Characterization of strains

The fermentation profiles of the mutants created was assessed during growth on ASYE medium in Falcon tubes as described in “Methods” section. In each case, three independent mutants of each generated strain were analysed. The comparative fermentation profiles of the genetically equivalent strains LS001 and TM89 and those of the strains LS003 and TM003 showed no significant differences (Fig. 4) and were in agreement with those previously reported by Cripps et al. [4]. However, much to our surprise, the solvent profile of the triple deletion mutant LS242 differed significantly from that of TM242, with only 20–30 mM ethanol achieved in 24 h compared to the levels (180–200 mM) attained by the strain TM242. It was further apparent that only 30% of the available glucose was consumed after 24 h in LS242, whereas all of the glucose was utilised by TM242 after an equivalent time period. These data suggest that glycolysis was impaired in LS242. Similar results were obtained for the same strain generated from TM89 (TM004) and in the two strains LS005 and TM005 in which an equivalent *pfl* ^−^ mutation to that present in TM242 was made, as opposed to the Δ*pfl* in-frame deletion created in LS242 and TM004. In both cases, however, the ethanol yields of LS005 and TM005 were about 0.1 g/g higher compared to the Δ*pfl* deletion variants, LS004 and TM004 (Table 2).

**Table 2:** Ethanol yield of the engineered strains of *G. thermoglucosidasius*

Strains	Metabolite concentration (mM) after 24 h of fermentation		
	Residual glucose	Ethanol	Ethanol yield (g/g)
NCIMB 11955	71.6	5.6	0.04 ± 0.01
LS001 (Δ*pyrE*Δ*ldh*)	80.7	37.7	0.32 ± 0.07
LS003 (Δ*pyrE*Δ*ldh pdh* ^up^)	44.3	95.2	0.37 ± 0.04
LS004 (Δ*pyrE*Δ*ldh pdh* ^up^ Δ*pfl*)	81.5	18.6	0.16 ± 0.02
LS005 (Δ*pyrE*Δ*ldh pdh* ^up^ *pfl* ^−^)	81.1	25.5	0.22 ± 0.12
LS242 (Δ*ldh pdh* ^up^ Δ*pfl)*	77.6	25.1	0.19 ± 0.04
TM89 (*ldh* ^−^)	89.0	37.7	0.44 ± 0.01
TM003 (Δ*pyrE ldh* ^−^ *pdh* ^up^)	41.8	84.9	0.31 ± 0.01
TM004 (Δ*pyrE ldh* ^−^ *pdh* ^up^ Δ*pfl*)	80.5	18.1	0.15 ± 0.05
TM005 (Δ*pyrE ldh* ^−^ *pfl* ^−^)	77.5	31.9	0.24 ± 0.02
TM242 (*ldh* ^−^ *pdh* ^up^ *pfl* ^−^)	0	186.6	0.43 ± 0.01
LS003^aa+^(Δ*pyrE*Δ*ldh pdh* ^up^ Δ*pfl*)	0	182.7	0.42 ± 0.04
TM003^aa+^(Δ*pyrE ldh* ^−^ *pdh* ^up^ Δ*pfl*)	0	180.4	0.42 ± 0.01
TM242^aa+^(*ldh* ^−^ *pdh* ^up^ *pfl* ^−^)	0	180.2	0.42 ± 0.01

^aa+^0.1 mM acetic acid added to culture medium

It has been suggested that by providing acetic acid in the media, the growth of Δ*ldh*Δ*pfl* mutants of two sub-strains of *G. thermoglucosidasius*, C56-YS93 and 95A1, could partially rescue growth during micro-anaerobic fermentation by providing the additional acetyl-CoA necessary for the regeneration of NAD^+^ [29]. We therefore tested whether a similar strategy could be applied here by supplementing the USYE media used for the growth of LS004 and TM004 with 0.1% acetic acid. The addition of acetic acid resulted in our strains having identical solvent profiles to that of TM242, with all of the available glucose utilised and the amount of ethanol produced equating to 180 mM, with a yield of 0.42 g/g. Compared with LS004 and TM242, which showed no significant increase or decrease of acetate concentration after fermentation (16.5 mM ± 0.144, 17.0 ± 0.429), a significant decrease of acetate level was observed with TM004. This suggests that 11955 is able to utilise acetate and significant differences are present between the strains.

### Genome analysis of selected mutant strains

**Fig. 5:**
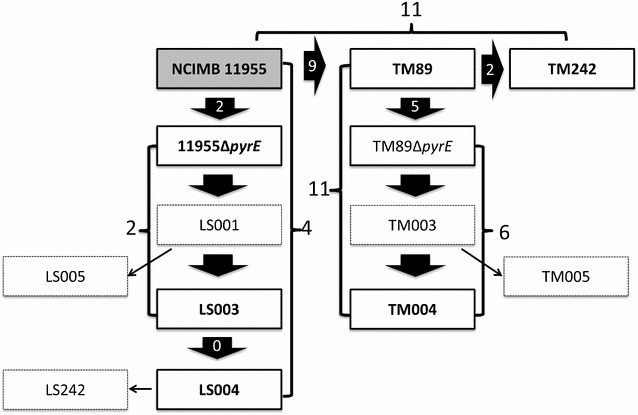
Overview of strain generation and SNPs acquired during strain generation. The construction of TM242 equivalent strains follows a stepwise path of *pyrE* mutant strain (Δ*pyrE)*, followed by *ldh* disruption, *pdh* up-regulation and *pfl* deletion both from NCIMB 11955 (11955Δ*pyrE,* LS001, LS003, LS005*)* and TM89 (TM003, TM004, TM005*)* progenitors. LS242 is the Δ*pyrE*-repaired strain of LS004. Strains in *solid black lines* were subjected to Illumina single-read sequencing and analysed by mapping the reads against the deposited genome of NCIMB 11955 (NCBI Accession Number: CP016622-CP016624) using CLCbio Genomic Workbench. Four mutations were acquired in the construction of LS004, two (1 Indel, 1 SNV) during the construction of 11955Δ*pyrE* and two (2 SNV) from 11955ΔpyrE to LS003. No mutation was acquired from LS003 to LS004. From TM89, a total of 11 mutations were acquired in TM004, with 5 (1 Indel, 4 SNV) during TM89Δ*pyrE* construction and 6 (all SNVs) from TM89*ΔpyrE* to TM004. A total of 9 differences were noted between TM89 and NICMB 11955 (1 Indel, 8 SNPs) with 2 further mutations found in TM242 (1 Indel, 1 SNV), resulting in a total of 11-nucleotide change compared to NCIMB 11955

To shed light on the possible reasons for the differences seen in the fermentation profile of the previously made TM242 and the equivalent strains made here, the genome DNA of selected strains was subject to Illumina paired-end sequencing and the sequence reads obtained mapped to the NCIMB 11955 reference genome [30] (NCBI accession number: CP016622–CP016624) using CLC Genomics Workbench. The analysed strains equated to NCIMB 11955*ΔpyrE*, LS003 (Δ*pyrE,* Δ*ldh, pdh* ^*up*^) and LS004 (Δ*pyrE*, Δ*ldh*, *pdh* ^*up*^, Δ*pfl*), TM89 (Δ*ldh*), TM89*ΔpyrE*, TM004 (Δ*pyrE*, *ldh* ^−^, *pdh* ^*up*^, Δ*pfl*) and TM242 (Fig. 5).

The results of the SNP/Indel analysis are shown in Table 3. From these data, it is clear that unintended mutations, in the form of single-nucleotide polymorphisms (SNPs) and insertions and deletions (Indels), occurred at each step of modification and once present were passed on to their progeny. During the previous construction of TM242 by Cripps et al. [4] from NCIMB 11955, a total of 11 unintended mutations had arisen. Most of these mutations (9) were present in the *ldh* mutant progenitor strain TM89. The equivalent strain to TM242 made here (strain LS004) from the wild-type NCIMB 11955 strain, using the developed *pyrE*-based allelic exchange system, carried 4 additional mutations compared to the parental strain 11955. Two of these mutations arose during the creation of the Δ*pyrE* strain needed for the mutagenesis method and two during the deletion of *ldh* and replacement of the *pdh* promoter. In comparison, a total of 11 mutations arose during the reconstruction of TM004. Five of these changes occurred during the derivation of TM89Δ*pyrE*, and six in the remaining two steps. Noticeably, the majority of the SNPs and Indels that had arisen occurred within coding regions. Moreover, most SNPs were non-synonymous causing changes in the encoded amino acid, and in the one case (LS003, position 71303) the introduction of a premature stop codon which truncated the encoded acyl-CoA dehydrogenase by 293 amino acids.

**Table 3:** Unique SNVs and Indels of strains used in this study compared to NCIMB 11955

Position	Strain	SNP	Gene	Locus	Effect
*Wild type progenitor*					
3448014	11955Δ*pyrE,* LS003, LS004	–>T	Heat-inducible transcription repressor HcrA	BCV53_17050	M184 fs
3791066	11955*ΔpyrE,* LS003, LS004	G>T	MenD	BCV53_17050	A496E
71303	LS003, LS004	G>A	Acyl-CoA dehydrogenase	BCV53_00405	Q292*
1329197	LS003, LS004	G>A	MFS transporter	BCV53_06565	–
*TM89 Progenitor*					
1037973	TM89, TM89*ΔpyrE, TM004, TM242*	A>G	L-rhamnose Isomerase	BCV53_05120	–
1466387	TM89, TM89*ΔpyrE, TM004, TM242*	C>T	N-acetyl-gamma-glutamyl-phosphate reductase	BCV53_07260	H210Y
1819652	TM89, TM89*ΔpyrE, TM004, TM242*	T>C	Transcriptional repressor codY	BCV53_09070	V143A
1842701	TM89, TM89*ΔpyrE, TM004, TM242*	A>T	Chemotaxis Protein CheA	BCV53_09205	N322 V
2844365	TM89, TM89*ΔpyrE, TM004, TM242*	T>C	Cytosolic protein	BCV53_13935	V1A
3210549	TM89, TM89*ΔpyrE, TM004, TM242*	T>−	30S ribosomal protein S1	BCV53_15740	N65 fs
3417349	TM89, TM89 *ΔpyrE, TM004, TM242*	A>G	Hypothetical protein	BCV53_16880	–
3515110	TM89, TM89*ΔpyrE, TM004, TM242*	C>T	Adenine phosphoribosyltransferase	BCV53_17395	D119 N
3515272	TM89, TM89*ΔpyrE, TM004, TM242*	T>G	Adenine phosphoribosyltransferase	BCV53_17395	T65P
1970245	TM89*ΔpyrE, TM004*	C>T	Hypothetical protein	BCV53_09795	R30C
1987439	TM89*ΔpyrE, TM004*	G>A	–	–	–
2792244	TM89*ΔpyrE, TM004*	C>T	ABC transporter permease	BCV53_13685	–
3281922	TM89*ΔpyrE, TM004*	C>–	pyrimidine-nucleoside phosphorylase	BCV53_16155	G96 fs
3412938	TM89*ΔpyrE, TM004*	C>A	–	–	–
651725	TM004	A>T	Gluconate: proton symporter	BCV53_03265	I155F
1671757	TM004	G>A	Transposase	BCV53_08315	Q167 K
2805178	TM004	T>A	Anti-anti-sigma factor	BCV53_13745	Q124D
3553391	TM004	G>T	Pilus assembly protein PilM	BCV53_17610	–
3759730	TM004	A>G	Leucine-tRNA ligase	BCV53_18565	Y540H
3760792	TM004	G>A	Leucine-tRNA ligase	BCV53_18565	P186S
34296*	TM242	A>–	–		–
2006731	TM242	G>A	Type III restriction modification system methylation subunit	BCV53_09980	E266 K

**Fig. 6:**
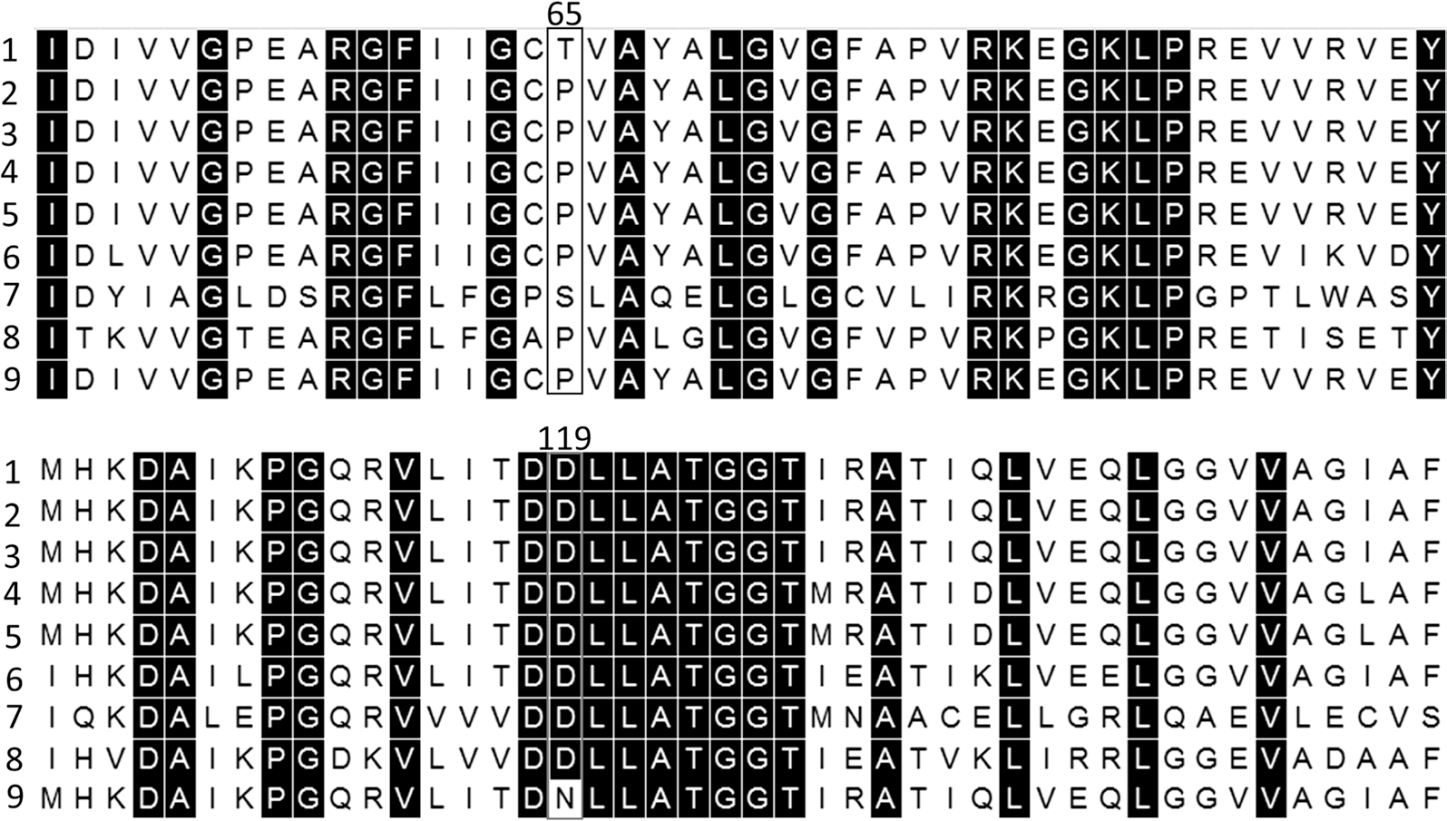
Adenosine phosphoribosyltransferase amino acid alignment. Amino acid sequences of adenosine phosphoribosyltransferase (APRT) from *G*. *thermoglucosidasius* NCIMB 11955 (*1*), DSM 2542 (*2*), C56-YS93 (*3*), G. *stearothermophilus* B4114 (*4*), *G. thermoleovorans* CCB_US3_UF5 (*5*), *B*. *subtilis* 168 (*6*), *Homo sapiens* (*7*), *E*. *coli* K-12 (*8*) and TM242 (*9*) are aligned in Mega using ClustalW, conserved sequences (100%) are highlighted in *black*. Positions 65 and 119 where SNPs occurred in TM89 and its derivative strains are indicated

A noticeable feature of the genome comparison data was the presence of two non-synonymous SNPs (T65P, D119N) in adenosine phosphoribosyltransferase (*aprt*) of TM89. Alignment of the *aprt* encoded protein sequence of five different thermophilic bacilli with homologues from *B. subtilis*, *E. coli* and *Homo sapiens* (Fig. 6) showed that while P65 is not conserved, the D119 residue forms part of absolutely conserved 9-amino acid region (DDLLATGGT). In a recent study [29], loss of *aprt* function in a mutant of *G. thermoglucosidasius* was shown to be due to the presence of a SNP (T123I) within this conserved domain. The same workers also demonstrated that the deliberate disruption of *aprt* improved both the growth and ethanol production rates in *G. thermoglucosidasius*. In view of the latter finding, strain LS006 (Δ*pyrE*Δ*ldhpdh* ^up^ Δ*pfl*Δ*aprt*) was constructed by making an in-frame deletion mutant of *aprt* in strain LS004. However, subsequent analysis of the strain revealed that it grew poorly during fermentation and was not able to utilise all of the available glucose, even with acetic acid supplementation.

## Discussion

In the present study, we have extended the application of our clostridial roadmap [6] for gene system development to *G. thermoglucosidasius*. We have designed and tested a series of *Geobacillus* vectors (pMTL60000 series) that conform to the highly successful pMTL80000 modular plasmid series [19] that are widely used in clostridia, in which the various vector components are localised to standardised modular parts, bounded by 8 nt restriction enzyme recognition sites. In terms of the replicons incorporated, it was desirable to have available defective derivatives to facilitate plasmid loss after homologous recombination. Here we used pUB110 and derivatives which replicate via a rolling circle mechanism [31]. Interference with the integrity of the replication regions of rolling circle plasmids can decrease the rate of conversion of ssDNA to dsDNA [32–34], which both affects plasmid stability and makes more recombinogenic ssDNA available. Indeed, in *B. subtilis*, recombination was shown to be stimulated 150- to 1500-fold in rolling circle plasmids compared with plasmids that do not generate single-stranded DNA [33]. Through the use different-sized regions of the pUB110 replicon, we showed that a 50-bp region between position −412 and −362 relative to *repB* played a significant role in plasmid stability. Those plasmids that retained this region, pTMO31 and pMTL61110, were 10-fold more stable (10^−3^ loss per generation) than those in which it was deleted (10^−2^ loss per generation), pMTL62110 or pMTL63110. The region may be part of the single-stranded origin (sso) [35, 36] or a membrane-binding site [37, 38], either contributing directly, or acting synergistically with other factors, to affect segregational stability. Fortuitously, the deletion of the 50-bp region had the additional benefit of decreasing the maximum temperature at which the plasmid could replicate from 60 to 55 °C. Temperature-sensitive (TS) plasmids, as demonstrated here, represent a particularly useful tool for modifying genomes [39]. In the protocol developed here, pure single-crossover integrants could be obtained after as little as 2 passages at 60 °C without the need for PCR screening.

In addition to the isolation of the TS plasmid, the other major step forward was the development of a counter-selection marker. Previously, laborious screening was required to isolate rare DC excision events from SC clones [4]. Numerous counter-selection systems have been reported [40, 41], including *pyrE/F* markers in the thermophilic bacteria *C. thermocellum*, *Clostridium bescii* and *G. kaustophilus* [14, 15, 42]. Interestingly, unlike in *G. thermoglucosidasius,* it proved necessary in the latter species to use both *pyrF* and *pyrR* to achieve the desired phenotype of 5-FOA resistance and uracil auxotrophy [15]. While most studies exploit the *pyrE/F* allele purely as a negative selection marker, ACE technology [16–18] also utilises *pyrE* alleles as positive selection markers by selecting for restoration of uracil prototrophy. Crucially, as the exploitation of *pyrE* as a counter-selection marker is reliant on the use of *pyrE* mutant host, ACE can be used to rapidly restore the *pyrE* allele to wild type, allowing any specific in-frame deletion mutant made to be characterised in a clean, otherwise wild-type background. The *pyrE* host may also be used to stably insert all manner of application-specific modules concomitant with correction of the *pyrE* allele. Here we exemplified this facility using the *pheB* gene (encoding catechol 2,3-dioxygenase), but theoretically DNA cargo of any size can be integrated.

For validation of the *pyrE*-based method developed, we sought to reconstruct TM242 [4], with the secondary aim of using the strains created as chassis for other useful products, such as* n*-butanol, isobutanol, succinate, etc. [43–45]. TM242 was made by making two in-frame deletions (*ldh* and *pfl*) and replacing a promoter region of a third gene (*pdh*) with an up-regulated variant [4]. From start to finish, the construction of the initial equivalent strain, LS242, took just 30 days. However, neither this strain, nor two subsequently independently derived equivalents, produced high titres of ethanol during microaerobic fermentation unless the medium was supplemented with acetate. A possible explanation for this is that the up-regulation of PDH might lead to insufficient NAD^+^ for the generation of acetyl-CoA, the precursor of ethanol [29]. By assimilating acetate through the action of either acetyl-CoA synthetase or acetate kinase/phosphate acetyltransferase [46], the cells may produce the levels of acetyl-CoA required for regeneration of NAD^+^ through the production of ethanol.

Although industrially acetate addition is not problematic due to its presence in lignocellulosic hydrolysate, failure to generate a TM242 equivalent strain prompted us to compare the genome sequences of the engineered strains. This analysis revealed that mutations, in the form of SNPs and Indels, were relatively common in the various strains sequenced. While there is a suggestion that 5-FOA is mutagenic in *Candia albicans* [47], this has not been observed when used with clostridial species [17, 18]. Moreover, the three unmanipulated reference strains sequenced, DSM 2542, NCIMB 11955 and TM89, all contain 4 or 5 unique SNPs unrelated to each other. Although there is not sufficient evidence to rule out possible mutagenic effects of 5-FOA, we speculate that the relatively high rate of mutation is a natural occurrence [48, 49], possibly related to the fact that the strain is a thermophile. Interestingly, between 4 and 8 SNVs/Indels have been reported to have occurred in *G. thermoglucosidasius* strains 95A1 and C56-YS93 after 10 time serial passages of the organism [29]. Taking into consideration the employed conditions, this equates to approximately 50 generations. Bearing in mind that the generation of a single in-frame deletion or insertion relies on the repeated isolation of colonies from solid media, each generated from a single cell in approx. 20 generations, the number of SNVs observed for our NCIMB 11955 triple and quadruple mutants suggests mutation rates which are comparable to those of 95A1 and C56-YS93, if not somewhat lower. Complex engineering of this and other species with similar mutation rates is likely to require improved screening and selection procedures to eliminate the accumulation of spontaneous off-target mutations. These could be based on the generation of multiple, independent strains for each step combined with whole-genome sequencing, an approach which is becoming feasible due to decreasing sequencing costs and increasing automation of routine steps in strain construction.

Due to the multiplicity of SNPs involved, it is not possible to determine why TM242 produces high titres of ethanol, whereas LS242 and related strains do not. TM242 could be overproducing ethanol as a consequence of mutations it has acquired, or LS242 and equivalent strains are not producing high ethanol titres because of mutations they have acquired. The *aprt* gene (encoding adenine phosphoribosyltransferase) could be the culprit as its deletion in a ***Δ*** *ldh* ***Δ*** *pfl* strain of *G. thermoglucosidasius* 95A1 brought about an increase in sugar consumption and ethanol production [29]. Moreover, TM242 does carry two SNPs within *aprt*, one of which is located in a conserved domain in which the acquisition of a different SNP by strain 95A1 led to the loss of *aprt* function [29]. However, the deliberate inactivation of *aprt* attempted here, through the creation of a knockout in LS004, still failed to produce the desired elevation in ethanol yields. One explanation would be that other mutations in the LS004 linage could be masking the effect of inactivation of *aprt.* One possible suspect is the mutation in the gene encoding the heat-inducible transcription repressor HcrA, which plays an important role in the regulation of stress response by controlling the level of chaperones [50]. Another is the gene encoding 2-succinyl-5-enolpyruvyl-6-hydroxy-3-cyclohexadiene-1-carboxylate (SEPHCHC) synthase (otherwise known as MenD), which is responsible for the first step of menaquinone biosynthesis, a key molecule involved in mediating electrons during anaerobic respiration, providing additional ATP through proton motive force [51].

The developed method represents a considerable improvement in the metabolic engineering tools available for use in *G. thermoglucosidasius* and thermophilic bacilli generally. The indicated timescale for making TM242 equivalents (30 days) represents the time it actually took and incorporates all stages required, including plasmid constructions, overnight growth of cells for competent cells and their preparation, streaking of isolated clones to purity and weekend rest days. For an individual mutational step, with the mutagenic plasmid at hand, the desired mutant may be generated within 5 days. Comparisons to other mutagenesis methods are not straightforward, as published protocols generally do not provide sufficient details. Estimates of two previously published methods for thermophilic bacilli [15, 52] suggest that mutant generation takes anywhere from 7 to 10 days. CRISPR/Cas9 genome editing has yet to be reported in *Geobacillus*, but two recent exemplifications in *B. subtilis* [53, 54] indicate that mutant generation is accomplished in 5–7 days.

## Conclusions

In the present study, we have extended the application of our clostridial roadmap to *G. thermoglucosidasius*. Through the use of ACE, a heterologous *pyrE* gene and a temperature-sensitive vector, a KO and KI system was developed with the respective turnaround times of 5 and 3 days and an efficiency of approximately 50%. As an exemplification of the method, strains equivalent to the industrial production strain, TM242 (two deletions and a promoter replacement), could be reproducibly generated in 30 days. Production of ethanol, however, in no case matched that of TM242 [4]. Genome sequencing of TM242, the parental strain NCIMB 11955 and the various mutant derivatives generated suggests that additional spontaneous mutations (SNPs and Indels) play a crucial role in the metabolic profile of the strains generated. This observation emphasises the need to routinely subject engineered strains to whole-genome sequencing.

## Methods

### Media, strain, plasmids and primers

Bacterial strains, plasmids and primers used in this study are listed in Tables 4, 5 and Additional file 1: Table SIII, respectively. 2SPY broth contained, per litre of deionised water, the following: soy peptone (Solabia or Sigma) (16 g), yeast extract (10 g), NaCl (5 g) and glycerol (10 g). 2SPYNG is 2SPY without glycerol. CBM, prepared according to O’Brien and Morris [55], consisted of, per litre of deionized water, MgSO_4_·7H_2_O (200 mg), MnSO_4_·H_2_O (7.58 mg), FeSO_4_·7H_2_O (10 mg),* p*-aminobenzoic acid (1 mg), biotin (2 μg), thiamine·HCl (1 mg), casein hydrolysate (acid hydrolyzed) (4 g), K_2_HPO_4_ (0.5 g) and KH_2_PO_4_ (0.5 g). USM medium was prepared according to Cripps et al. [4].

**Table 4:** Bacterial strains used in this study

Strain	Genotype	Reference/source
*E. coli* Top 10	F-*mcrA ∆ (mrr*-*hsdRMS*-*mcrBC) Φ80lacZ∆M15 ∆lacX74 deoR recA1 araD139 ∆ (ara*-*leu) 7697 galU galK rpsL (StrR) endA1 nupG*	Invitrogen
NCIMB 11955		TMO
DSM 2542		DSM
TM242	*ldh* ^−^ *pfl* ^−^ *pdh* ^*up*^	TMO
DSMZ 7263		DSM
DSMZ 5366		DSM
11955Δ*pyrE*	Δ*pyrE*	This study
LS001	Δ*pyrE*Δ*ldh*	This study
LS003	Δ*pyrE*Δ*ldh pdh* ^*up*^	This study
LS004	Δ*pyrE*Δ*ldh* Δ*pfl pdh* ^*up*^	This study
LS005	Δ*pyrE*Δ*ldh pfl* ^−^ *pdh* ^*up*^	This study
LS242	Δ*ldh* Δ*pfl pdh* ^*up*^	This study
TM89	*ldh* ^−^	TMO
TM89Δ*pyrE*	Δ*pyrE ldh* ^−^	This study
TM003	Δ*pyrE ldh* ^−^ *pdh* ^*up*^	This study
TM004	Δ*pyrE ldh* ^−^ *pdhp* ^*u*^ Δ*pfl*	This study
TM005	Δ*pyrE ldh* ^−^ *pdh* ^*up*^ *pfl* ^−^	This study
LS006	Δ*pyrE ldh* ^−^ *pdh* ^*up*^ *pfl* ^−^ Δ*aprt*	This study

**Table 5:** Plasmids used in this study

Plasmid	Relevant description	Source
pTMO31	*G. thermoglucosidasius* shuttle vector, pMB1, pUB110, Amp^R^, Kan^R^	TMO
pNW33 N	*G. thermoglucosidasius* shuttle vector, pMB1, pUB110, Cm^R^	BGSC
pUC31	*G. thermoglucosidasius* shuttle vector, pMB1, pUB110, Kan^SR^	TMO
pMTL85151	Clostridial modular shuttle vector, ColE1+*tra*, pIM13, Cm^R^	This study
pMTL62110	*G. thermoglucosidasius* modular shuttle vector, ColE1+*tra*, pUB110, Kan^R^	This study
pMTL63110	pMTL86551 with shorter pUB110	This study
pMTL61110	pMTL86551 with longer pUB110	This study
pMTL-LS1	Vector for generation of *pyrE* mutant via allelic exchange	This study
pMTL-LS2	*pyrE* mutant correction vector	This study
pMTL-LS3	Vector for integration of DNA at *pyrE* locus via allelic exchange	This study
pMTL-LS3::pheB	Vector for integrating *pldh::pheB* at the *pyrE* locus	This study
pMTL-LS5	Complementation vector for *pyrE* mutant based on *pyrE* from *G. kaustophilus*	This study
pMTL-LS6	Complementation vector for *pyrE* mutant based on *pyrE* from *G. thermoleovorans*	This study
pMTL-LS5::*pfl*	*pfl* in-frame deletion vector	This study
pMTL-LS5::*ldh*	*ldh* in-frame deletion vector	This study
pMTL-LS5::*ldhp*:*pdh*	*pdh* promoter replacement vector with *ldh* promoter from *G. stearothermophilus*	This study
pMTL-LS5::*pfl*242	*pfl* deletion vector allowing disruption in the same context as that of TM242	This study
pMTL-LS5::*aprt*	*aprt* in-frame deletion vector	This study
pMTL-LS5::*trpB*	*trpB* in-frame deletion vector	This study

### Growth conditions and transformation

*Geobacillus* strains were grown as appropriate at 52–61 °C on TSA plate or in 2SPYNG/2SPY media shaken at 250 rpm supplemented with appropriate antibiotics. Transformation of the strains, as well as characterizations for ethanol and organic acid production in 50-ml Falcon tubes, was conducted in accordance with the procedure described by Cripps et al. [4].

### Analytical methods

Growth of all bacterial cultures was monitored by measuring optical density at 600 nm (OD_600_) using a Pharmacia Novaspec II. Supernatant samples from Falcon tube fermentation were subjected to HPLC analysis using the method described by Cooksley et al. [56] with slight modification. Ethanol, acetate, pyruvate, lactate, formate and glucose were measured using a Thermo Scientific HPLC (Ultimate 3000) with a Phenomenex Rezex ROA-organic acid H + (8%) 150 × 7.8 mm (P/N: 00F-0138-K0; S/N: 514195-27) column. Samples were kept at 4 °C before passing through the column at a flow rate of 0.5 ml/min, with column temperature at 35 °C UV_VIS_1 detector wavelength at 210 nm, seal washed with water:methanol (90:10) for 30 min. The mobile phase used was 0.005 M H_2_SO_4_ with 50 mM valerate as an internal standard.

### Construction of pMTL60000 series modular shuttle vector

The *Clostridium* pMTL series shuttle vector [19] was used to construct pMTL61110. The kanamycin acetyltransferase gene was amplified with primers kanF and kanR from either pTMO31 or pUC31 [25] and cloned between the *Fse*I and *Pme*I sites of pMTL85151, replacing its chloramphenicol resistance module with kanamycin resistance. The Gram-positive replicon module, defined by *Asc*I and *Fse*I sites, was then replaced with different variants of pUB110, amplified also from pTMO31 using primers RepB_F, RepB_F2 and RepB_F3 with RepB_R as fragments of different lengths, yielding pMTL61110 (4809 bp), pMTL62110 (4591 bp) and pMTL63110 (4418 bp).

### Construction of pMTL-LS1—the *pyrE* knockout vector

An internal 300-bp *pyrE* fragment 3 bp from its 5′ end lacking only the start codon was amplified from *G. thermoglucosidasius* 11955 genomic DNA using primers PyrELHA_F/R and cloned into the *Sbf*I and *Not*I sites of pMTL62110. This is followed by the insertion of a 1200-bp PCR fragment with primers PyrERHA_F/R starting from the stop codon of *pyrE* into the HindIII/AscI site yielding pMTL-LS1.

### Construction of pMTL-LS2—the *ΔpyrE* correction vector

Primers PyrE_LHA_F and PyrE_RHA_R were used to PCR amplify the homology fragment comprising the whole *pyrE* gene lacking the start codon and continued downstream by the 1200-bp RHA. Ligation of this 2379-bp fragment generated into the AscI and SbfI sites of pMTL62110 yielded the *pyrE* correction vector pMTL-LS2.

### Construction of pMTL-LS5—the *pyrE* complementation vector

Heterologous *pyrE* gene needed to complement strain 11955Δ*pyrE* was amplified with a set of primers designed based on the genomic sequences of *G. kaustophilus* HTA425 and *G. thermoleovorans* CCB_US3_UF5 obtained from NCBI using genomic DNA extracted from strains DSM 7263 and DSM 11667 as template. The fragments were then TA-cloned into the Invitrogen topo-2.1 vector. The *pyrE* genes were fused to the 3′ end of *kan* in pMTL86551 through overlap extension PCR using primers Comp_PyrE_F/R and Comp_Kan_F/R. The resultant product is the complete kanamycin gene, including its native promoter, linked to the *pyrE* gene of *either G. kaustophilus or G. thermoleovorans,* separated by a 26-bp untranslated region, containing the RBS sequence TGAAGGAGGATGAATGCA, with 9 bp between the start codon and the SD sequence. Ligation of this dual-selective marker fragment between the FseI and PmeI sites of pMTL62110 yielded the complementation plasmids pMTL-LS5 and pMTL-LS6.

### Construction of in-frame deletion vectors

Marker-less in-frame deletion vectors for *ldh*, *pfl, aprt* and *trpB* knockout and replacement of *pdh* promoter with *G. stearothermophilus ldh* promoter were all based on pMTL-LS5. Using overlap extension PCR with appropriate primers, left and right homology arms, ~500 bp each, corresponding to up- and downstream of the genes containing just the start and stop codons, were amplified and fused together and inserted in the MCS of pMTL-LS5, yielding pMTL-LS5::*ldh*, pMTL-LS5::*pfl,* pMTL-LS5*::aprt and* pMTL-LS5*::trpB.* To construct the *pdh* promoter replacement vector, a 1176-bp promoter replacement cassette, consisting of a 156-bp *ldh* promoter fragment flanked by 500 bp upstream of *pdh*, and 500 bp encompassing the 5′ PDH complex E1 coding region, was synthesised as 4 gBlocks by Integrated DNA Technologies with 30-bp overlaps between the fragments. Blocks 1 and 4 also overlapped with two ends of linearized pMTL-LS5 cleaved by *Hind*III and *Xba*I, allowing assembly via NEB Gibson cloning, thereby yielding pMTL-LS5::*ldhp*:*pdh.*

### Construction of pMTL-LS3—the *pyrE* integration vector

To generate the *pyrE* integration vector, a 642-bp PCR product which is composed of the entire *pyrE* gene lacking only the start codon was amplified with primers PyrE_LHA_F and PyrE_Int_R using *G. thermoglucosidasius* 11955 genomic DNA and cloned in the *Sbf*I and *Not*I sites replacing the LHA in pMTL-LS1, yielding pMTL-LS3.

### Plasmid segregational stability assay

Plasmid segregational stability was assessed using a modified method previously described [57]. Briefly, *G*. *thermoglucosidasius* strain 11955 was transformed with plasmids containing different replicons and selected on TSA plate with antibiotic. The following day, single colonies of each transformant were picked and inoculated at 52 °C for 16 h in 10 ml 2SPYNG with antibiotics. Then 100 μl of the cultures was used to inoculate 10 ml 2SPYNG again with antibiotics at 52 °C for 12 h. From this point on, the cells were inoculated as mentioned previously but in non-selective 2SPYNG for 12 h. This was repeated for 72 h at every 12-h interval. After each 12 h of inoculation without selection pressure, serial dilutions were carried out for each broth from 10^−1^ to 10^−7^ in fresh 2SPYNG pre-warmed for 30 min at 52 °C. A 100-μl aliquot of each dilution was plated out on non-selective TSA plate pre-dried for 1 h at 37 °C. The following day, one hundred single colonies from the TSA plates were replica plated using a 1-μl sterile inoculation loop on TSA plates with and without antibiotic. After 24 h, all colonies were counted against a grid and the percentage of plasmid loss was calculated using the difference between the number of non-resistant and resistant colonies. Plasmid retained per generation was calculated with the equation $\sqrt[n]{R}$ and plasmid lost per generation as $1 - \sqrt[n]{R}$, where n is the number of generation and R is the percentage of cell population retaining the plasmid.

### Phenotypic assay of PheB

*Geobacillus thermoglucosidasius* transformed with *pheB*-based reporter constructs was re-streaked on TSA plate with appropriate antibiotics. After 24 h of cell growth, the cells were spread with 100 mM catechol. The *pheB* gene product, catechol 2,3-dioxygenase (C23O), catalyses the dioxygenolytic metacleavage of the catechol aromatic ring to yield HMSA, which gives a vivid yellow colour.

### Next-generation sequencing and analysis

Genomic DNA for next-generation sequencing was prepared by phenol chloroform extraction from the strains DSM 2542, NCIMB 11955, TM89, TM242, NCIMB 11955*ΔpyrE*, LS003, LS004, TM89*ΔpyrE* and TM004. Paired-end libraries were prepared and sequenced (251 bp reads) using an Illumina MiSeq at DeepSeq, University of Nottingham. Preparation of paired-end libraries as well as sequencing was performed as described by the manufacturers. Reads were analysed using CLC Genomics Workbench and Artemis. SNPs/Indels were called using a cut-off threshold frequency of 80% with quality-based variant detection. For lineage analysis, alignment of the SNPs/Indels callings for the different strains based on reference locus was performed on Microsoft Excel. Selected SNVs and Indels were confirmed by amplifying a few hundred base pairs up- and downstream of the area of interest and the amplicons were Sanger sequenced (Source Bioscience, UK) (Primers in Supplementary Materials). The genome sequences of NCIMB 11955 and TM242 are deposited at GenBank under the accession numbers of CP016622-CP016624 and CP016916-CP016918, respectively.

## Additional file

**Additional file 1.** This file contains all additional tables and figures.

## Abbreviations

5-FOA: 5-fluorouracil
5-FUMP: 5-fluorouridine monophosphate
5-FI: 5-fluoroindole
ACE: allele-coupled exchange
APRT: adenine phosphoribosyltransferase
DC: double crossover
HMSA: 2-hydroxymuconic semialdehyde
Indels: insertions and deletions
Km: kanamycin
KI: knock-in
KO: knock-out
LHA: left homology arm
MCS: multiple cloning site
PDH: pyruvate dehydrogenase
RHA: right homology arm
SC: single crossover
SNP: single-nucleotide polymorphism
SNV: single-nucleotide variation
