# Supplementary material for: Development and implementation of rapid metabolic engineering tools for chemical and fuel production in Geobacillus thermoglucosidasius NCIMB 11955
Source: Biotechnol Biofuels. 2017 Jan 3;10:5. doi: 10.1186/s13068-016-0692-x (PMC5210280; doi:10.1186/s13068-016-0692-x)
Supplement: Supplementary file 1 — Additional file 1. This file contains all additional tables and figures. [file 13068_2016_692_MOESM1_ESM.docx]

**Supplementary Material**

**Development and implementation of rapid metabolic engineering tools for chemical and fuel production in *Geobacillus thermoglucosidasius*** **NCIMB 11955**

Lili Sheng, Klaus Winzer, Ying Zhang and Nigel P. Minton*

Clostridia Research Group, BBSRC/EPSRC Synthetic Biology Research Centre (SBRC), School of Life Sciences, University of Nottingham, University Park, Nottingham NG7 2RD, UK.

**Tables**

**Table SI: Uracil requirement for Δ*pyrE* mutant generated in this study**

| **Strain** | **Uracil Concentration (µg/ml)** | | | | | | | |
| --- | --- | --- | --- | --- | --- | --- | --- | --- |
|  | 0 | 1 | 2 | 5 | 10 | 15 | 20 | 40 |
| **11955** | +++ | +++ | +++ | +++ | +++ | +++ | +++ | +++ |
| **TM89** | +++ | +++ | +++ | +++ | +++ | +++ | +++ | +++ |
| **11955Δ*pyrE*** | - | + | + | ++ | +++ | +++ | +++ | +++ |
| **TM89Δ*pyrE*** | - | + | + | ++ | +++ | +++ | +++ | +++ |

**Table SII: Nucleotide sequence identity of different Geobacillus *pyrE* genes compared to NCIMB 11955 *PyrE***

| **Strain** | **Sequence Identity** |
| --- | --- |
| *Geobacillus sp.* WCH70 | 81% |
| *Geobacillus sp.* Y412MC52 | 77% |
| *Geobacillus sp.* Y412MC61 | 77% |
| *Geobacillus sp.* GHH01 | 77% |
| *Geobacillus thermodenitrificans* NG80-2 | 77% |
| *Geobacillus kaustophilus* HTA426 | 77% |
| *Geobacillus sp.* C56-T3 | 76% |
| *Geobacillus thermoleovorans* CCB_US3_UF5 | 75% |

**Table SIII. Primers used in this study**

| **Primer Name** | **Sequence (5’ – 3’)** |
| --- | --- |
| Kan_F | CACCGGCCGGCCATTATTGCAATGTGGAATTGGGAACGG |
| Kan_R | CACCGTTTAAACGTGCACCATATATGCGGTGTGAAAT |
| pUB110_F1 | CACCGGCGCGCCGTTTGTAGACAAGGTAAAGGATAAAACAGCA |
| pUB110_F2 | CACCGGCGCGCCGATGGTTTTGAACTTGTTCTTTCTTATCTT |
| pUB110_F3 | CACCGGCGCGCCACTGGCCGTCGTTTTACAACGT |
| pUB110_R | CACCGGCCGGCCGCATCCTTCAATCCTTTTAATAACAATTATAGC |
| PyrE_LHA_F | CCTGCAGGGAAGGGAGAAGAGAAAGAATGAAAAAAG |
| PyrE_LHA_R | GCGGCCGCGCGCACATAGCACATCGGC |
| PyrE_RHA_F | AAGCTTTAAAATCAACGCAAGCGAAAAGGAGC |
| PyrE_RHA_R | GGCGCGCCGGCTTATTATGTTGCAACGGAACATAGT |
| PyrE_Int_R | GCTACGGCGGCCGCTTATTTCCCCCACTTTTCCGGGT |
| PyrE_C1_F | CCCATGCTGAAAATCCAGCTG |
| PyrE_C1_R | GCCGCTGTATCCATATGACCA |
| PyrE_C2_F | GCCTGCAGACATGCAAGC |
| PyrE_C2_R | CGGGTCGACAGAATTGTTCG |
| Trp_LHA_F | CGGCCCTGCAGGAACGTTCCAAACGAAAACGGCAG |
| Trp_LHA_R | CGGCGCGGCCGCCCGTTTAGCGAGCAGTGCC |
| Trp_RHA_F | CGGCGCTAGCGCCGATGTTTATCCCGTTTATTGTT |
| Trp_RHA_R | CGGCGGCGCGCCGCGGAATTTCACGGATTTCCG |
| PFL_UpF | ACTGGATCCGCAGTTCCGCAAACGCTTAAG |
| PFL_UpR | GATTATTACATCGTTTCGTGTTGTTTCATAACAGTTTCCCTCCCA |
| PFL_DownF | GGGAAACTGTTATGAAACAACACGAAACGATGTAATAATCTCCC |
| PFL_DownR | ACTGGTACCAGTTTCCGGTGTTTTTTCTCATCGA |
| LDH_UpF | ACTGGATCCCATCGCGTCGTCCGCTATAT |
| LDH_UpR | GTTGTATTCAAAGTCAGTATTATTGTTGTTTCATCGCTGTCTGTCA |
| LDH_DownF | CAGCGATGAAACAACAATAATACTGACTTTGAATACAACAAGGTG |
| LDH_DownR | ACTGGTACCGGCCGCTTTTAATGGATGAAATCCT |
| FC_Trp | GAAAAGTGGAAGTGGAGACGGTAA |
| RC_Trp | AATTCGTGCAATGTTTCCGGCAGT |
| FC_PLF | GGACATGGGCGATTATTTTGCTTTC |
| RC_PLF | GCCGGATCCAAACAGGAACG |
| FC_LDH | GATGTACAACATCAGCAACTACGG |
| RC_LDH | CAATAACCGGAATTCCTATCGCTC |
| PDH_C1_F | GCAGCGGTTTATCTGGTTGAC |
| PDH_C1_R | ATTCATCCTCCCTCAATATAATGCG |
| PDH_C2_F | ATGGAAATAAGGTACCGCGGG |
| PDH_C2_R | GATGCCAGGAATTCCCGCT |
| APRT_LHA_F | GATGGAATTCGCTGTTGGATATGCCTCCGTC |
| APRT_LHA_R | GTAAGCTCAATCAAAAAGGGAAATCAGGAACAATTGTCACATATTGTTTC |
| APRT_RHA_F | GACAATTGTTCCTGATTTCCCTTTTTGATTGAGCTTACCGAGC |
| APRT_RHA_R | GATGCTCGAGGGCGGGATCCATTTTTAAATCGAC |
| APRT_CF | CGTTAGGAATCGGAGCATATAAAG |
| APRT_CR | CGACAACATCGTGCAGAAATCC |
| FC_Plasmid | GACGCGTGACGTCGACTCT |
| RC_Plasmid | AACAGCTATGACCGCGGCC |
|  |  |

**Table SIV – Primers for validation of all SNVs and Indels that occurred in this study**

| **SNP locus** | **Sequence (5’ – 3’)** |
| --- | --- |
| 34269 | **F:** GCCTAAAAATGCGCCAAGACAAAC **R:** GTCAGAAGTAAATTAGTTTCTCGTCGCTCC |
| 71303 | **F:** GAACTAACAGGCGGTTGATTTCG **R:** CGGATTTGCCGATGTGTTTATCG |
| 651725 | **F:** GGCATGGTTCCTGCCATCTTG **R:** CGATTGGAAGGGCGATCATAGGAT |
| 1037973 | **F:** CATTGTTGGCAAGGTGATGATGTTCG **R:** CTGAACCAATTCCAAACAGCTTACTTTC |
| 1329197 | **F:** GCTGATTCTCATTGCCATGTTATTGTTGG **R:** CTCAAGCTGTTCCAAAAGCGTTGC |
| 1466387 | **F:** CTAATGAGCATGAAGTCGTCTTTTTTGCC **R:** GACGGAAACAACCGTTACTCTTCCC |
| 1671757 | **F:** GATTTCCGCACCATCAACCGATTTC **R:** CTAAATCCAGTGATGAATTGGTTCCCTG |
| 1819652 | **F:** GATTAAGCAGTCGATTGAAAATGAACGGATG **R:** CCGGTTTCTTTATGATCCATTAACTGGATTTC |
| 1842701 | **F:** CGCTGAAGAATTATACGAACGGATTATGAAAG **R:** CAATGCCATGGTCAATCGCATTGC |
| 1970245 | **F:** CGGATAATTAAATCTCTTGTAATTCGCGAAGC **R:** GCATCTGTCGAATAAGAGGAAATTCCG |
| 1987439 | **F:** CGTCAAACTGCGCCCATATTGTG **R:** CGTGCTCTGTTTGCTTGATTCGG |
| 2006713 | **F:** CGACAACCTCGAAGTATTAAAGCTG **R:** CTGGTCTTTGATCATCGGACATGAT |
| 2792242 | **F:** GTAAATACTATAAACGCAATGACAAGCGCC **R:** CATGTTTATCGAAAATGCGATCGATTTGTTTC |
| 2805178 | **F:** CCACTATCAGCTCAATGAACATAAGCAAC **R:** GTTAGTTTACAATACACAACAACTGGCGAG |
| 2844365 | **F:** GCGGATTATTATAGACCAATATCTGCATTGC **R:** GTTTGTCCGACGCCATCCTG |
| 3210549 | **F:** GAGGGAGACACGACCGTTCTC **R:** GTTTCTGTCGGCGATGAAGTGAC |
| 3281992 | **F:** CGTATCAATCGCTTCTTTCACTTCG **R:** GTCAAGGGAATTATCGCAGAAATGG |
| 3412938 | **F:** CGTCGGCATCGGTGCTATCTTAAG **R:** CATTATTGTCGGATTTATTGCACGCC |
| 3417349 | **F:** GCGTTAAGAAGAATGACAAGAGTCGC **R:** CAGTTCTTTAACCCATTTTTTTCTTTCTTGCG |
| 3448014 | **F:** GATACCATTTGGTCAACACGGTC **R:** CGGATTAATCCAATCATGTCGATGG |
| 3515110 | **F:** CGGATTAATCCAATCATGTCGATGG **R:** CGCTTGAAACAAGCTCAGTATGAG |
| 3515272 | **F:** CGGATTAATCCAATCATGTCGATGG **R:** CGCTTGAAACAAGCTCAGTATGAG |
| 3553391 | **F:** GATCGTCTTTCTTCACTTGATCCGC **R:** GACATTGTGGAATGGTATCAGCAAATG |
| 3759730 | **F:** GACGACATTTCCTCTCGATTTGC **R:** GGTTAGCTTATATTGATGAAGTTCCG |
| 3760792 | **F:** GACGACATTTCCTCTCGATTTGC **R:** GGTTAGCTTATATTGATGAAGTTCCG |
| 3791066 | **F:** CAATATTTCTTCCAGATCAGCAGCC **R:** GTTATTTGTCGGCAACAGTATGCC |

**Figures**


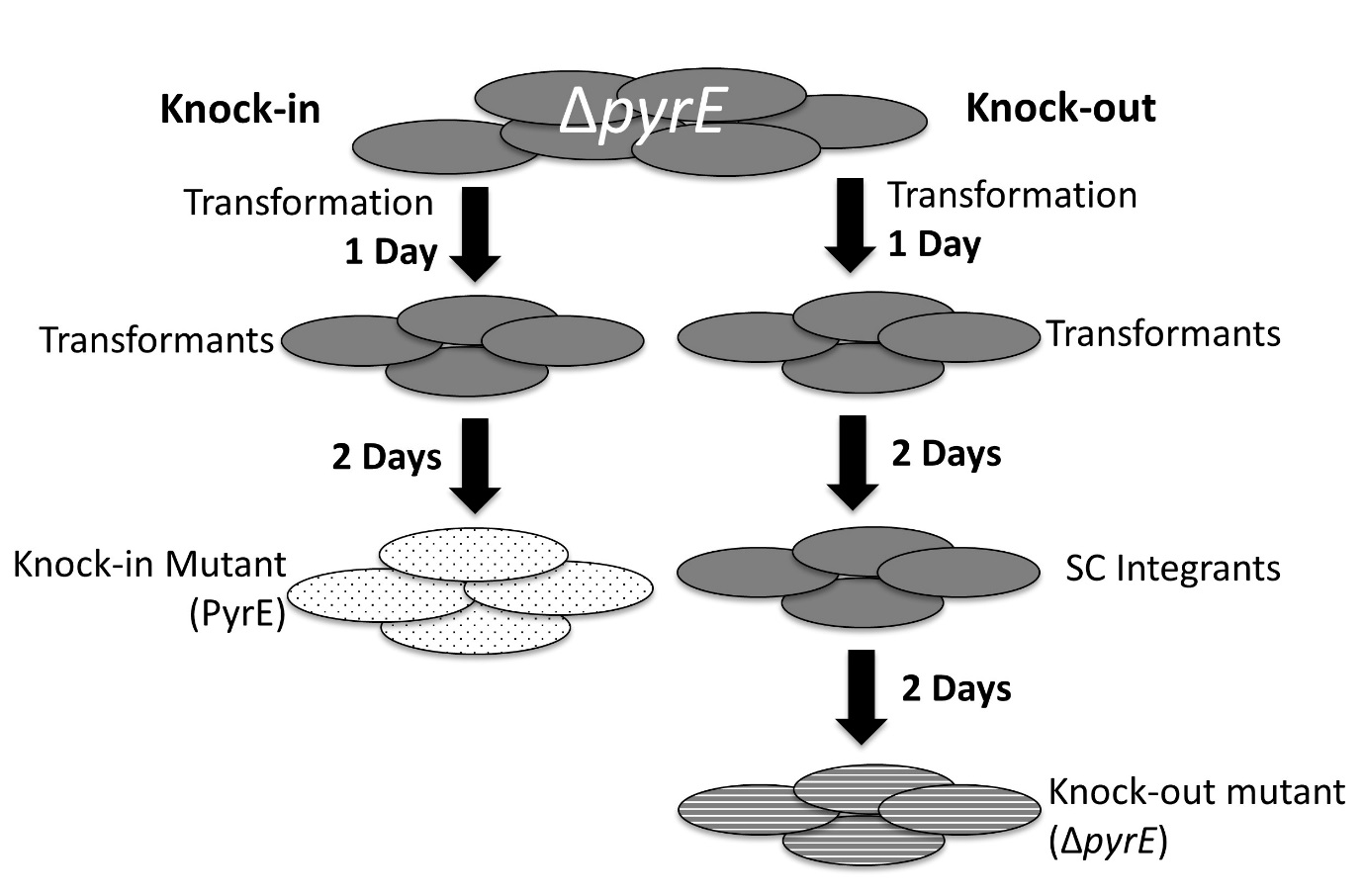


**Figure SI: Schematic diagram showing the timeline of knock-in and knock-out in *G. thermoglucosidasius* using ACE.** Strains in *ΔpyrE* genotype are transformed with appropriate plasmids for knock-in and knock-out. Knock-in: successful transformants selected on kanamycin are passaged on to uracil deficient medium, typically, 2 passages are adequate for obtaining pure double crossover (DC) mutant. Plasmid pMTL-LS2 repairs the *ΔpyrE* genotype to WT *pyrE* while pMTL-LS3 allows integration of heterologous DNA. Knock-out: Successful transformants selected on kanamycin are passaged on to uracil deficient medium, typically, 2 passages are adequate for obtaining pure single crossover (SC) integrant. The SC mutants are further screened with 5-FOA to obtain DC mutant (requiring only 1 passage), which can either revert to wild type or result in the desired knock-out. After PCR verification, the retained *ΔpyrE* genotype can be paired to WT *pyrE,* or integrated with DNA following the knock-in procedure, either for complementation of the deleted gene or for construction of heterologous pathways.

**
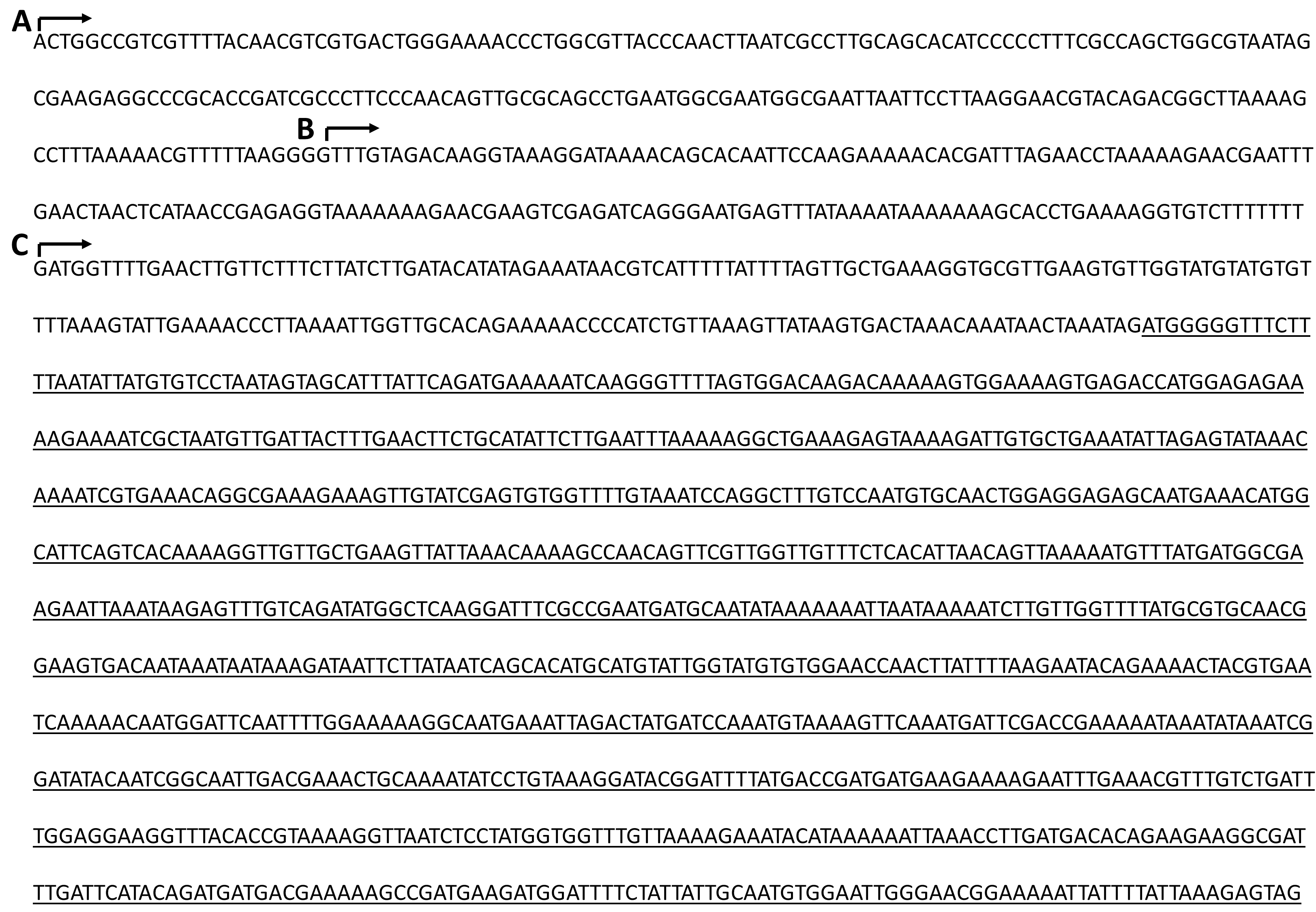
**

**Figure SII: Sequence of pUB110 replicon and its variants.** The coding sequence of the replication protein B is underlined. A, B and C represent the start of the 5’ incompatibility region and the two foreshortened variants, giving rise to pMTL61110, pMTL62110 and pMTL63110 respectively.

**
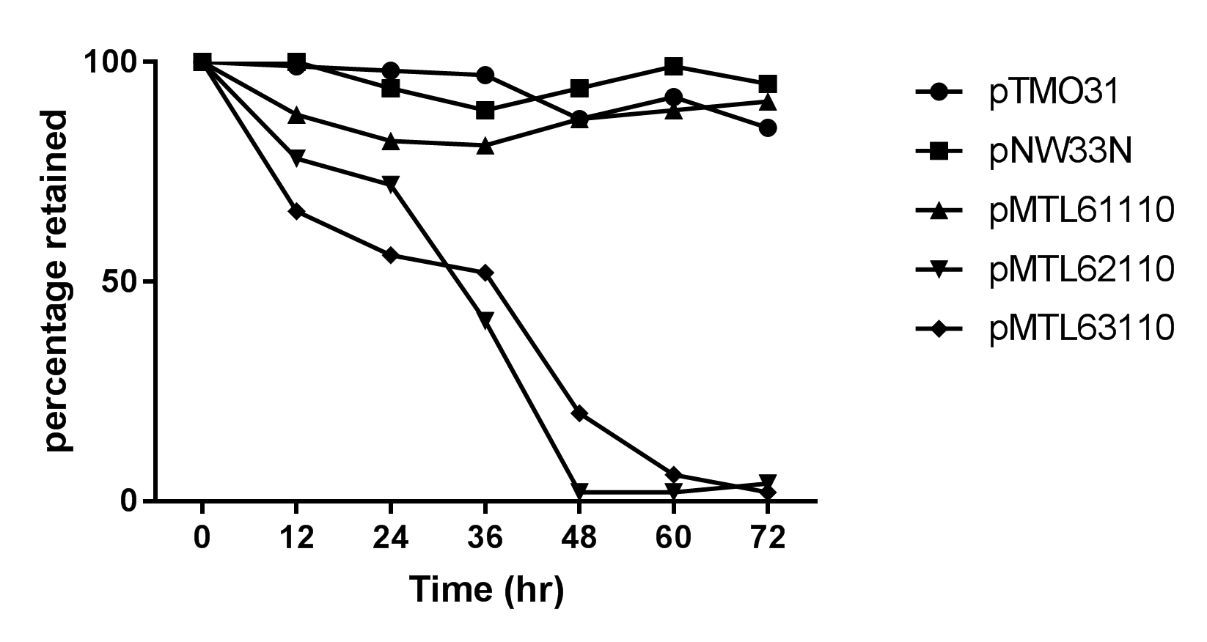
**

**Figure SIII: Plasmid retained by cells at 52 °C.** Transformants of *G*. *thermoglucosidasius* NCIMB 11955 were picked and inoculated at 52 °C for 16 h in 10 ml 2SPYNG with antibiotics. Then 100 μl of the cultures were used to inoculate 10 ml 2SPYNG again with antibiotics at 52°C for 12 h. This allowed the maintaining of the plasmids while getting cells to the same state. From this point on, cells were inoculated like previous but to 10 ml of non-selective 2SPYNG for 12 h. The action was repeated for 72 h at every 12 h interval. After each 12 h of inoculation without selection pressure, serial dilutions were carried out for each broth from 10^-1^ – 10^-7^ in fresh 2SPYNG pre-warmed for 30 min at 52° C. A 100 μl aliquot of each dilution was plated out on non-selective TSA plate pre-dried for 1 h at 37° C. On the following day, one hundred single colonies from the TSA plates were replica plated using a 1 μl sterile inoculation loop on TSA plates with and without antibiotic. Colonies were counted and the percentage plasmid loss was calculated using the difference between number of non-resistant and resistant colonies.

**
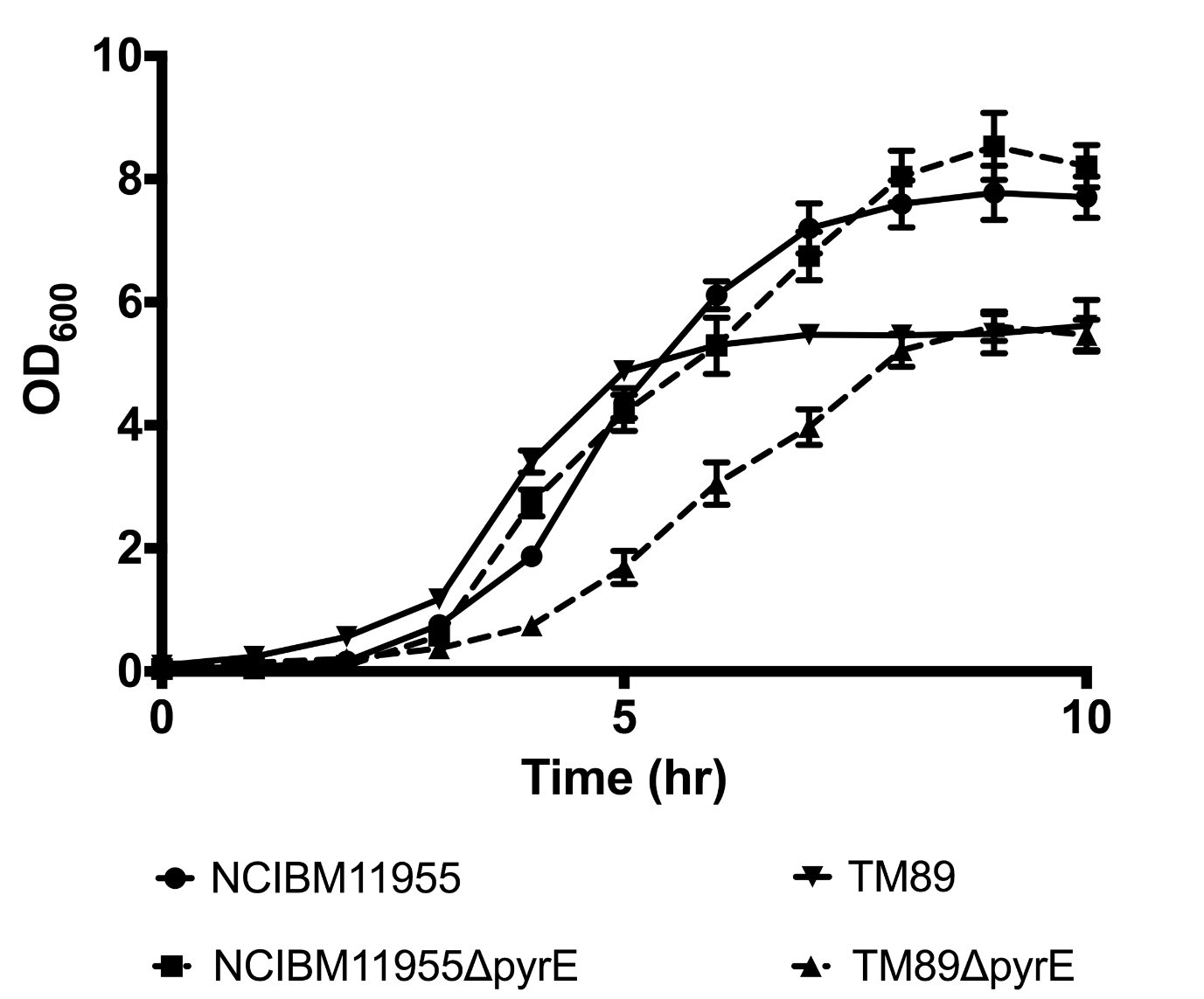
**

**Figure SIV – Growth curve of G. *thermoglucosidasius* NCIMB 11955 and TM89 and their corresponding *pyrE* mutants.** Strains (3 biological replicates) are grown in 2SPYNG medium and OD_600_ are recorded every h.


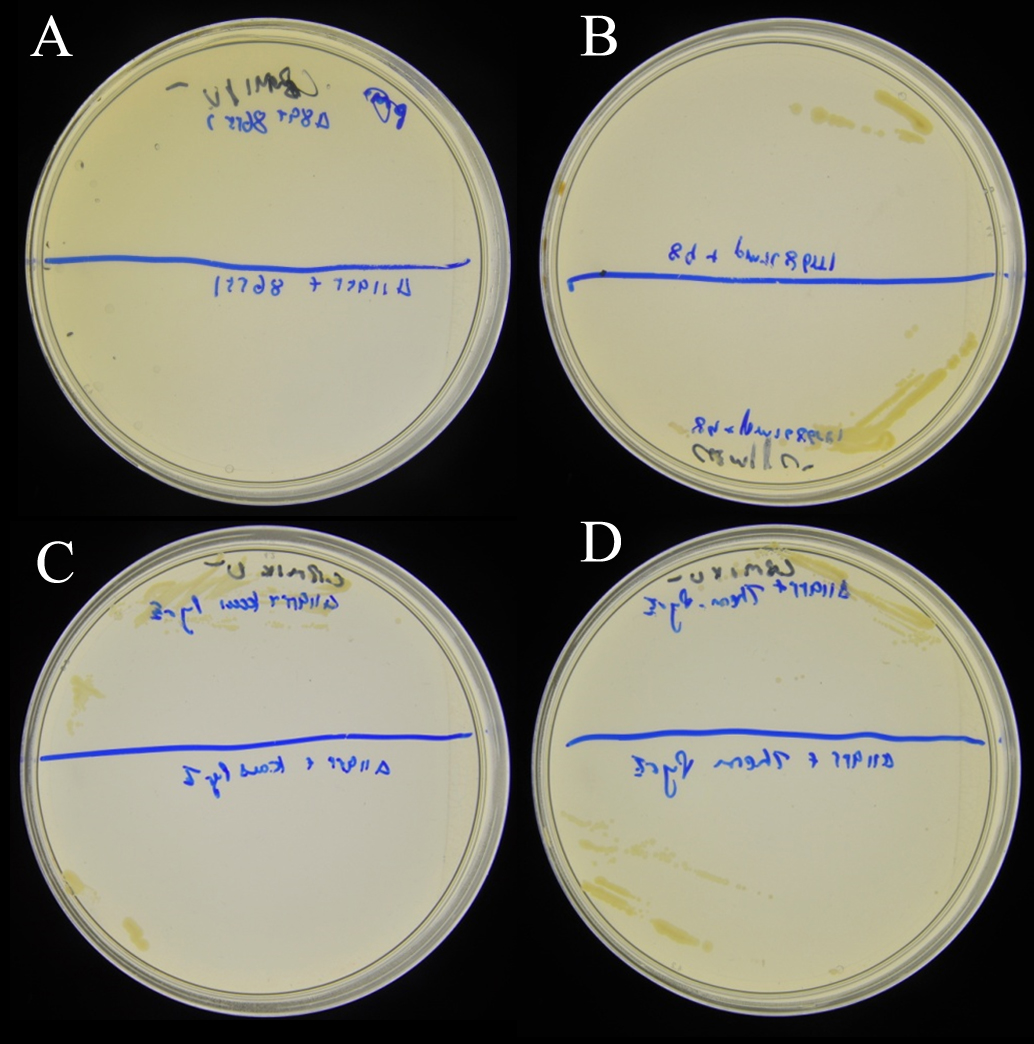


**Figure SV: Complementation of uracil autotrophy of 11955*ΔpyrE* with pMTL-LS5 and pMTL-LS6.** Two colonies per transformation were re-streaked onto CBM1X uracil deficient plate and photos were taken after incubation at 52 °C for 24 h. A: 11955*ΔpyrE* with pMTL62110 (positive control – no growth); B: TM89 with pMTL62110 (negative control - growth); C: 11955*ΔpyrE* with pMTL-LS5 (complemented – growth); D: 11955*ΔpyrE* with pMTL-LS6 (complemented – growth)


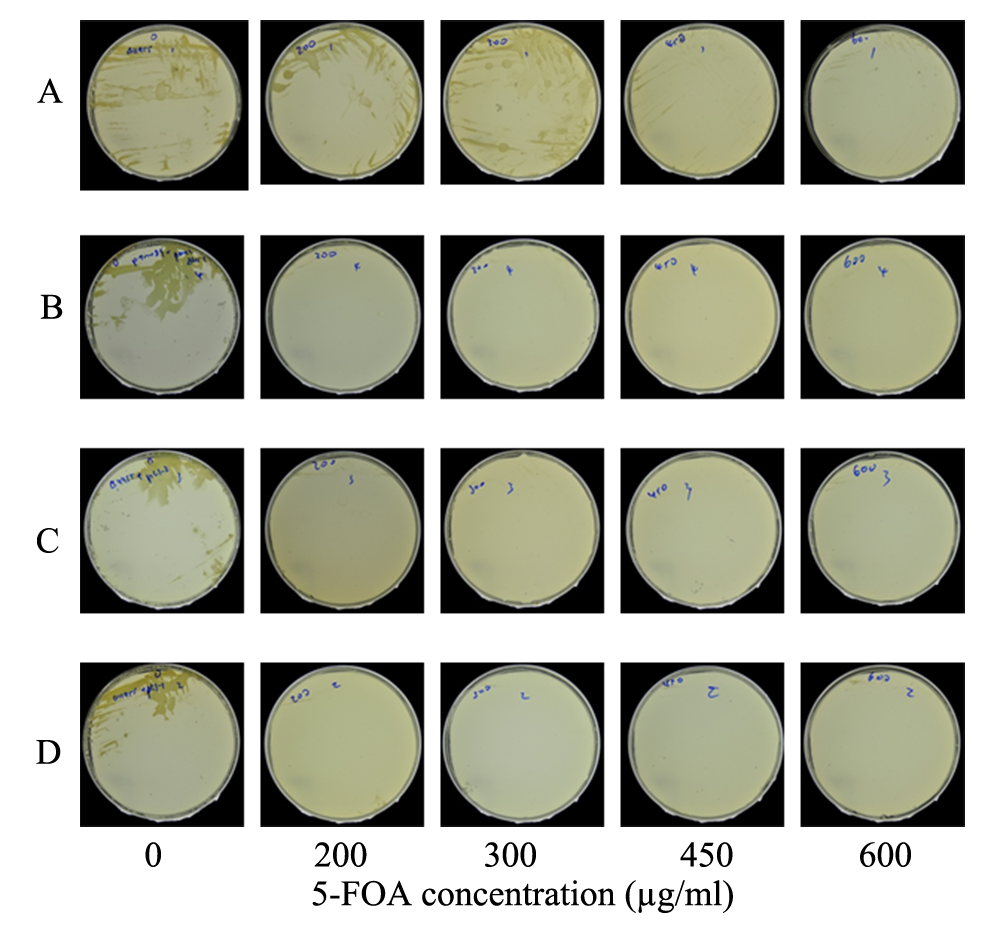


**Figure SVI: Complementation the 5-FOA toxicity with pMTL-LS5 and pMTL-LS6 for NCIMB 11955 *pyrE* mutant.** Two colonies per transformation were re-streaked onto CBM1X containing 5-FOA at different concentrations supplemented with 20 µg/ml uracil and photos were taken after incubation at 52 °C for 24 h. The *ΔpyrE* strain (A) were resistant to 5-FOA at up to 600 µg/ml. Wild type strain (B) and *ΔpyrE* strain with plasmids pMTL-LS5 (C) and pMTL-LS6 (D) failed to grow at 5-FOA concentration of 300 µg/ml.


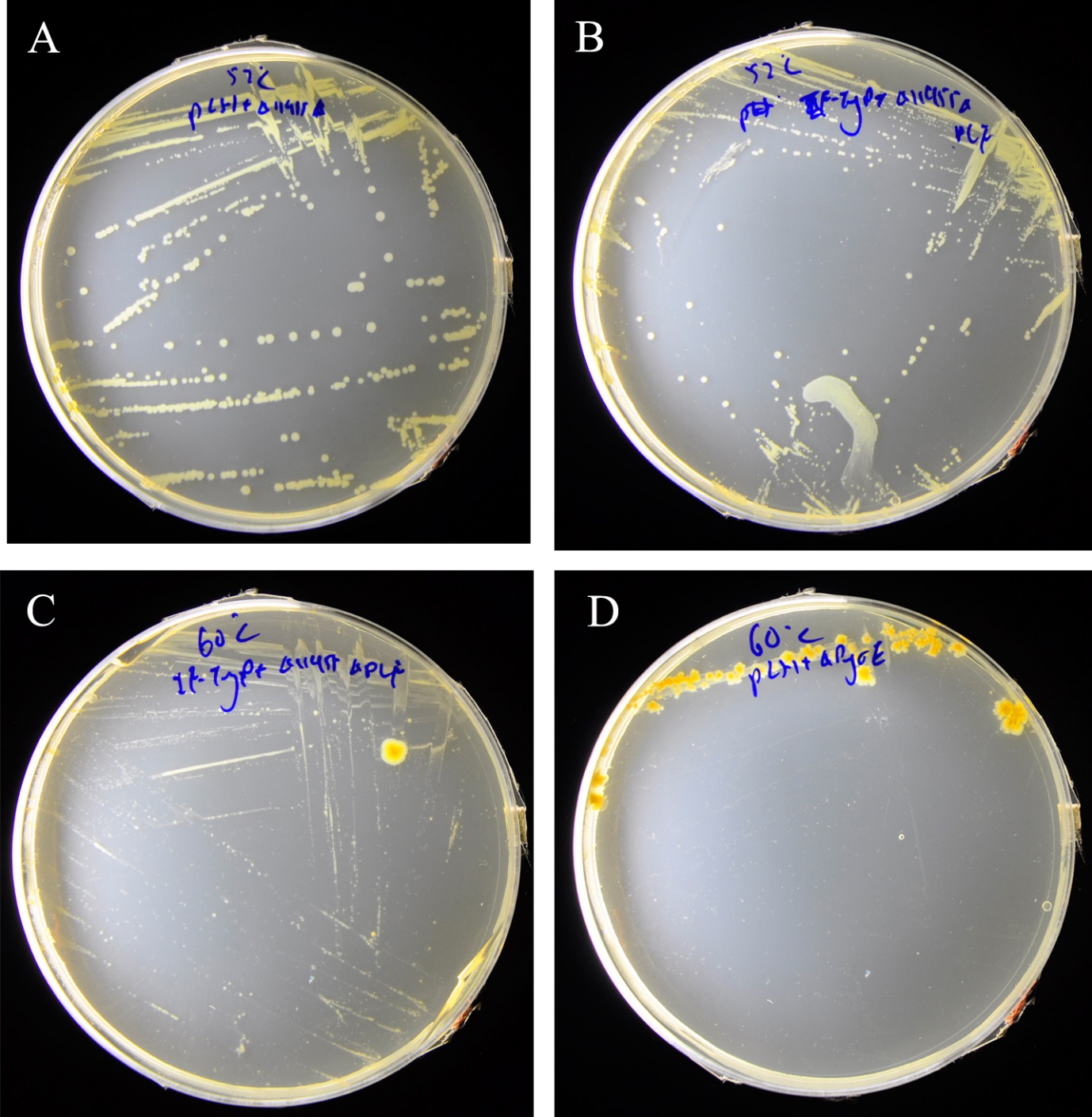


**Figure SVII: Temperature and uracil based selectivity for single crossover mutants.** In the absence of uracil, NCIMB 11955*ΔpyrE* is complemented with pMTL-LS5 at 52 °C with (B) and without (A) region of homology to *G. thermoglucosidasius* NCIMB 11955 chromosome. At 60 °C, 11955*ΔpyrE* can only grow with region of homology (C). No discrete colony appeared without region of homology (D).
